# Omega-3 fatty acid DHA induces ferroptosis in colorectal cancer patient-derived organoids and drug-tolerant cells

**DOI:** 10.1038/s41419-026-08744-8

**Published:** 2026-04-11

**Authors:** Laura di Blasio, Marianela Vara-Messler, Barbara Peracino, Elena Masti, Vanesa Cepas-López, Livio Trusolino, Alberto Puliafito, Andrea Bertotti, Valentina Monica, Luca Primo

**Affiliations:** 1 Department of Oncology, University of Torino, Torino, Italy; 2 Candiolo Cancer Institute, FPO-IRCCS, Candiolo, Italy; 3 Department of Clinical and Biological Sciences, University of Torino, Torino, Italy; 4 Present Address: Sanofi Belgium, Zwijnaarde, Belgium

**Keywords:** Fatty acids, Gastrointestinal cancer, Cancer models, Cancer metabolism, Cancer stem cells

## Abstract

Several epidemiological and preclinical studies suggest that omega-3 (n-3) polyunsaturated fatty acids (PUFAs) exert anticancer activity at multiple stages of colorectal cancer (CRC) progression. However, inconsistent clinical evidence and the lack of a clearly defined molecular mechanism underlying the antitumor effects of n-3 PUFAs have raised doubts about their efficacy as anticancer therapies. To address these issues, we investigated the effects of the n-3 PUFA docosahexaenoic acid (DHA) in a collection of CRC patient-derived tumor organoids (PDTOs), a powerful platform for functional analysis of patient-specific tumors. DHA treatment markedly reduced CRC cell viability in a time- and concentration-dependent manner without inducing apoptosis. CRC-derived PDTOs exhibited pronounced sensitivity to DHA, irrespective of KRAS or TP53 mutational status, whereas organoids from normal colon tissue were less affected. Mechanistically, DHA induced ferroptosis in both CRC cells and PDTOs, as evidenced by lipid peroxide accumulation and partial rescue by ferroptosis inhibitors. Fluorescently labeled DHA localized predominantly to the endoplasmic reticulum and mitochondria, where it promoted oxidative stress. Moreover, DHA impaired the regrowth of oxaliplatin-tolerant persister cells and enhanced oxaliplatin efficacy in sequential treatment models. Together, these findings indicate that exploiting the intrinsic oxidative vulnerability of cancer cells with DHA may represent a promising, low-toxicity strategy to enhance chemotherapy efficacy and target drug-tolerant persister cells in colorectal cancer.

## Introduction

Ferroptosis is a regulated, iron-dependent form of non-apoptotic cell death characterized by the accumulation of lethal lipid peroxides [1]. This process is triggered when lipid peroxidation surpasses the capacity of cellular antioxidant systems [2, 3]. Polyunsaturated phospholipids, particularly those containing polyunsaturated fatty acid (PUFA) chains, are highly vulnerable to peroxidation at bis-allylic sites [4]. Long-chain n-6 and n-3 PUFAs, such as arachidonic acid (ARA; C20:4), eicosapentaenoic acid (EPA; 20:5) and docosahexaenoic acid (DHA; C22:6), are especially prone to oxidation due to their high degree of unsaturation and, as a result, serve as potent inducers of ferroptosis [5].

Although long-chain n-3 PUFA can be synthesized from shorter essential n-3 fatty acids (FA), as linolenic acid, this metabolic pathway is not very efficient in humans, so much of it is derived from the diet. PUFAs are important fatty acids for membrane fluidity by being incorporated into membrane phospholipids, and in addition, they serve as substrates for synthesis of specialized pro-resolving mediators that actively turn inflammation off [6, 7].

Beyond their role in physiological functions, n*-*3 PUFAs can affect some chronic diseases such as cancer. In particular, epidemiological and preclinical evidence suggest that n-3 PUFAs have activity in several stages of colorectal cancer (CRC) management, from prevention to advanced metastatic disease [8]. Worldwide, CRC is a leading cause of mortality and morbidity and the third most common cause of death from cancer [9]. Most CRC cases are sporadic and develop in a step-wise manner known as adenoma-carcinoma sequence characterized by accumulation of mutations in different signaling pathways that leads to the initiation and progression of CRC [10].

While early-stage CRC can often be cured by surgery, metastatic disease remains largely incurable due to the persistence of disseminated tumor cells and is therefore treated with systemic therapies. Combination chemotherapy represents the backbone of metastatic CRC treatment, often combined with targeted agents such as EGFR inhibitors in molecularly selected patients [11]. However, these treatments rarely achieve complete tumor eradication, as drug-tolerant persister cells frequently survive and drive disease relapse, underscoring the need for therapeutic strategies able to eliminate residual resistant tumor populations [12].

Several studies have also considered the potential therapeutic activity of n*-*3 PUFAs against established solid tumors in addition to preventive effects [13–15]. In a phase III randomized observational trial in CRC patients, a higher n-3 PUFA intake was associated with improved 3-years disease free-survival for KRAS wild-type tumors [16], while in a phase II interventional trial a pre-operative treatment with EPA provided postoperative overall survival benefit [17]. Still, the current state of evidence in human studies shows contrasting outcomes that need clarification [18]. Indeed, the high degree of inter-individual variability in metabolizing fatty acids may explain in part the inconsistent results from clinical trials. An ongoing phase III trial administering EPA in the pre- and post-operative setting is expected to provide more comprehensive insights into the impact of PUFAs in the treatment of CRC [19].

To overcome these limitations, we took advantage of the ground breaking technology of patient-derived tumor organoids (PDTOs), which maintain intra-tumor phenotypic cell diversity, as well as patient genetic heterogeneity [20]. PDTOs have enormous potential for therapy development and precision medicine providing an unprecedented opportunity for functional studies on tumors from individual patients [21, 22]. Our collection of PDTOs is mainly derived from liver metastases of colorectal cancer previously expanded in mice and is completely molecularly and functionally characterized [23]. We also generated a collection of PDTOs from primary colorectal tumors and their healthy colon counterparts. These models enable to directly observe cell and metabolic modifications induced by drugs, but also to compare tumor and normal organoids identifying side-effects and drug-mediated toxicity events.

While a number of biological effects that could contribute to the anti-cancer activity of n-3 PUFA have been previously described, the exact mechanism exploited by n-3 PUFA to inhibit cancer growth has not been unveiled yet [24]. It is known that n-3 PUFAs are highly susceptible to peroxidation, so that PUFA levels in the membrane must be precisely controlled. Indeed, an increase in membrane PUFA peroxidation can trigger cell death via ferroptosis [1]. Recently, it has been demonstrated that the amount of diacyl-PUFA phosphatidylcholines in cells is a marker of ferroptosis sensitivity and drives ferroptosis through the initiation of reactive oxygen species (ROS) production in mitochondria and lipid peroxidation [25].

Here we show that treatment with DHA inhibits the growth of colorectal cancer cells and PDTOs, including the most aggressive KRAS mutated tumors, by inducing ferroptosis. This effect on cell viability is much less evident in non-tumoral organoids. Moreover, DHA markedly impairs the regrowth capacity of oxaliplatin-persister cells in PDTOs, highlighting its potential to eliminate drug-tolerant cells (DTC) responsible for tumor relapse.

## Results

### DHA treatment inhibits colorectal cancer cells growth inducing non-apoptotic cell death

**Fig. 1:**
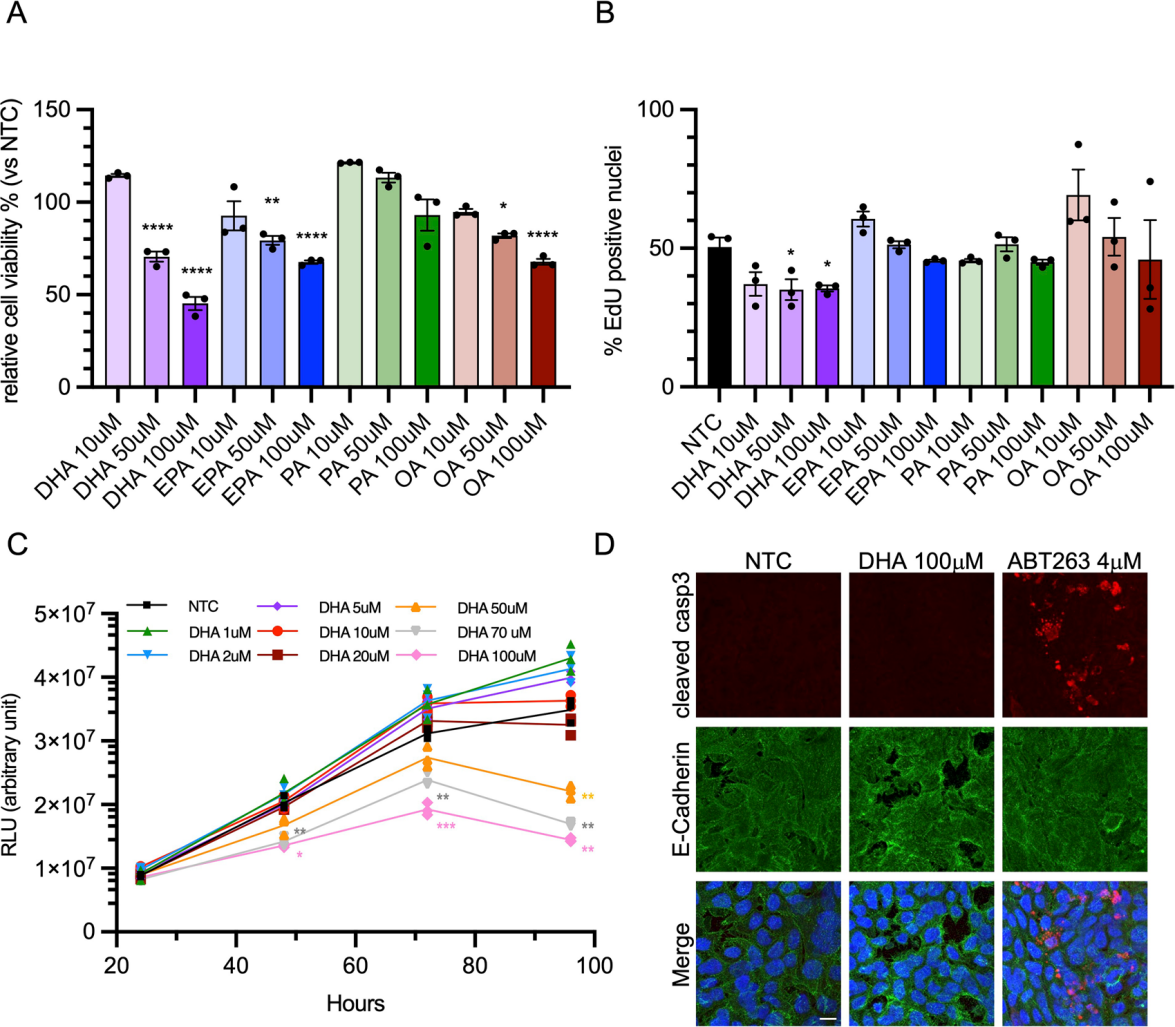
DHA treatment inhibits colorectal cancer cells growth inducing non-apoptotic cell death. **A** HT29 were treated twice at 48-h interval with DHA, EPA, PA and OA (10 μM, 50 μM and 100 μM), or BSA as control; viability was measured as ATP content after a total of 72 h. The percentage of viable cells in different conditions compared to BSA-treated control (NTC) is plotted as mean ± SEM; each dot corresponds to the average of one independent experiment; *p < 0.05, **p < 0.001, ***p < 0.001, ****p < 0.0001 *versus* NTC. **B** HT29 were treated twice at 48-h interval with DHA, EPA, PA and OA (10 μM, 50 μM and 100 μM), or BSA as control; EdU was added to cells for the last 6 h of treatment. Cells were fixed and stained after a total of 72 h. The percentage of EdU-positive nuclei in each condition is plotted as mean ± SEM; *p < 0.05 *versus* NTC. **C** HT29 were treated with DHA at 1, 2, 5, 10, 20, 50, 70, 100 μM, or BSA as control, at 48-h interval; viability was measured as ATP content after 24, 48, 72, and 96 h from the first treatment. The luminescence measured at each time point is plotted as mean ± SEM; each dot corresponds to the average of one independent experiment; statistical significance: DHA 70 μM *versus* NTC at 48 h **p < 0.01; DHA 100 μM *versus* NTC at 48 h *p < 0.05; DHA 70 μM *versus* NTC at 72 h **p < 0.01; DHA 100 μM *versus* NTC at 72 h ***p < 0.001; DHA 50, 70, 100 μM *versus* NTC at 96 h **p < 0.01. **D** HT29 were treated for two consecutive days with DHA (100 μM), ABT263 (4 μM) as positive control or BSA as negative control; after 48 h cells were fixed and stained with cleaved Caspase 3 (red), E-Cadherin (green) and NucBlue™; scale bar 10 μm.

We first compared how different FAs affected growth and viability of colorectal cancer cells. We evaluated the effect of saturated fatty acids (palmitate, PA), mono-unsaturated fatty acids (oleate, OA) and n-3 PUFAs (EPA and DHA), on the cell line HT29, a widely studied model of colon cancer growth. The addition of PA did not significantly affect cell growth compared with cells treated with BSA alone (not-treated control; NTC). In contrast, EPA and OA induced a modest but statistically significant reduction in cell viability at 50 µM, reaching approximately 70% viability at 100 µM relative to untreated control. By comparison, DHA treatment was particularly effective at inhibiting cell growth, with a significant reduction already observed at 50 µM and a decrease in cell viability exceeding 50% at 100 µM. (Fig. 1A).

To determine whether the reduction in cell growth was due to impaired cell replication, we assessed EdU incorporation in the cells (Figs. 1B and S1). Treatment with DHA slightly reduced cell replication but not in a dose-dependent manner. Conversely, no significant differences were observed with EPA and PA. Interestingly, the addition of low doses of OA appeared to even stimulate cell replication, even if not in a significant manner. Then, to examine the cytotoxic effects of DHA, we evaluated the cell response in time-course experiments, expanding the range of tested concentrations. DHA treatment was effective starting from 48 h, with a marked increase in cytotoxicity observed after 96 h. Notably, this effect displayed a concentration threshold at 50 μM, as lower concentrations were completely ineffective. (Fig. 1C). This finding highlights the existence of a concentration- and time-dependent threshold for DHA-induced cytotoxicity.

The reduction in cell viability prompted us to investigate whether DHA activated an apoptotic cascade. The evaluation of active Caspase-3 level ruled out apoptotic cell death (Fig. 1D). These findings suggest that DHA reduces the growth of CRC cells without inducing apoptosis, but rather through a distinct cytotoxic mechanism.

### DHA induces ferroptosis in colorectal cancer cells

**Fig. 2:**
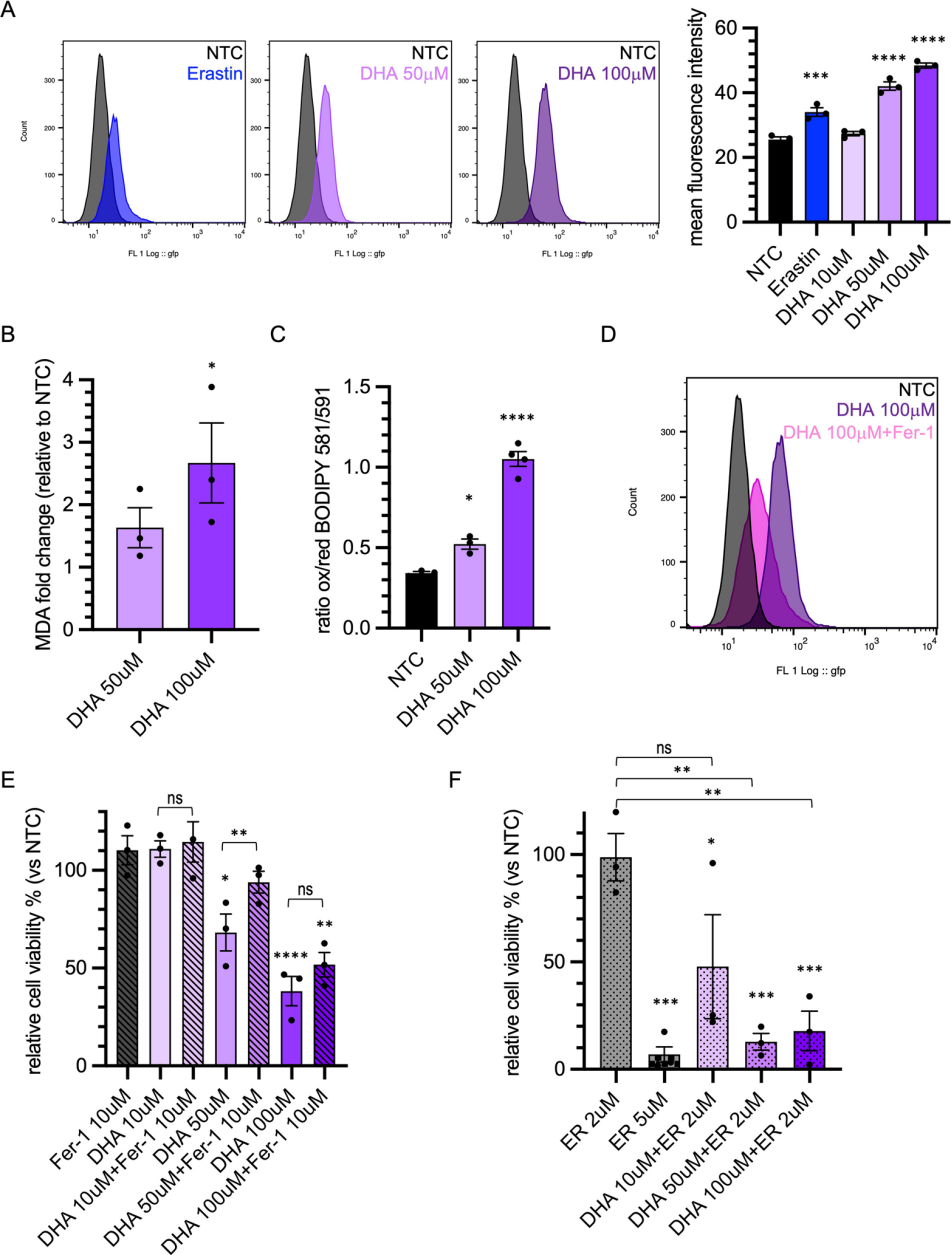
DHA induces ferroptosis in colorectal cancer cells. **A** HT29 were treated twice at 48-h interval with DHA (10, 50 and 100 μM), Erastin (5 μM) as positive control or BSA as negative control; lipid peroxidation was detected after a total of 72 h using Liperfluo and analized by flow citometry. Representative histograms are shown; mean fluorescence intensity from 3 independent experiments is plotted as mean ± SEM; each dot corresponds to the average of one independent experiment; ***p < 0.001, ****p < 0.0001 *versus* NTC. **B** HT29 were treated twice at 48-h intervals with DHA (50 and 100 μM), or BSA as negative control, then after 72 h lipid peroxidation was measured using the Lipid Peroxidation MDA Assay kit. Malondialdehyde fold change (nmol/well) produced in each condition is plotted as mean ± SEM; *p < 0.05 *versus* NTC. **C** HT29 were treated twice at 48-h interval with DHA (50 and 100 μM) or BSA as negative control; after 72 h Image-iT® lipid peroxidation kit was used to visualize lipid peroxidation. The ratio between mean fluorescence intensities of the dye at 590 nm (oxidized) and 510 nm (reduced) was plotted as mean ± SEM; *p < 0.05, ****p < 0.0001 *versus* NTC. **D** HT29 were treated twice at 48-h interval with DHA (100 μM) alone or in combination with Ferrostatin-1 (Fer-1, 20 μM), BSA was used as negative control; lipid peroxidation was detected after a total of 72 h using Liperfluo and analysed by flow cytometry. Representative histogram is shown. **E** HT29 were treated twice at 48-h interval with DHA (10 μM, 50 μM, 100 μM) alone or in combination with Fer-1 (10 μM), BSA was used as control; after 5 days, viability was measured as ATP content and plotted as percentage of viable cells (mean ± SEM) in different conditions compared to BSA-treated control (NTC). Statistical significance: DHA 50 μM *versus* NTC *p < 0.05; DHA 100 μM *versus* NTC ****p < 0.0001; DHA 100 μM + Ferrostatin-1 *versus* NTC **p < 0.01; DHA 10 μM *versus* DHA 10 μM + Ferrostatin-1 ns; DHA 50 μM *versus* DHA 50 μM + Ferrostatin-1 **p < 0.01; DHA 100 μM *versus* DHA 100 μM + Ferrostatin-1 ns. **F** HT29 were treated with Erastin (2 and 5 μM) alone or in combination with DHA (10 μM, 50 μM, 100 μM) with the same schedule described above; viability was measured as ATP content and plotted as percentage of viable cells (mean ± SEM) in different conditions compared to BSA-treated control (NTC). Statistical significance: DHA 10 μM + Erastin 2 μM *versus* NTC *p < 0.05; Erastin 5 μM, DHA 50 μM + Erastin 2 μM and DHA 100 μM + Erastin 2 μM *versus* NTC ***p < 0.001; Erastin 2 μM *versus* DHA 10 μM + Erastin 2 μM ns; Erastin 2 μM *versus* DHA 50 μM + Erastin 2 μM **p < 0.01; Erastin 2 μM *versus* DHA 100 μM + Erastin 2 μM **p < 0.01.

Since DHA-induced cell death does not appear to be apoptotic, we hypothesized that DHA treatment may trigger a ferroptotic process. Ferroptosis is primarily characterized by lipid peroxidation, a process sustained by the presence of PUFAs. Lipid peroxidation levels in cells increased significantly following treatment with 50 µM and 100 µM DHA, reaching levels higher than those induced by Erastin, a well-established ferroptosis inducer (Fig. 2A). Notably, lipid peroxides accumulation appeared to be concentrated within the cell in discrete, yet poorly defined compartments (Fig. S2A). These findings were supported by the results of the malondialdehyde (MDA) assay (Fig. 2B), which highlighted an elevated amount of lipid peroxidation following DHA treatment, as well as by the use of the lipid peroxidation-sensitive fluorophore, BODIPY 581/591 C11 (Figs. 2C and S2B). Moreover, lipid peroxidation level was reduced when cells were treated with both DHA and the ferroptosis inhibitor ferrostatin-1 (Fer-1) (Fig. 2D and S2C). Even DHA-induced cell death was partially recovered when cells were treated with Fer-1 (Fig. 2E). Conversely, the administration of DHA together with Erastin markedly enhanced cell death, even at concentrations of 10 µM DHA, usually insufficient to induce any cytotoxic effects. Furthermore, the combined treatment with DHA made Erastin effective at inducing cell death at a concentration as low as 2 µM (Fig. 2F).

These observations support the hypothesis that DHA actively contributes to ferroptosis-mediated cell death.

### DHA is incorporated into subcellular compartments and induces mitochondrial oxidative stress

**Fig. 3:**
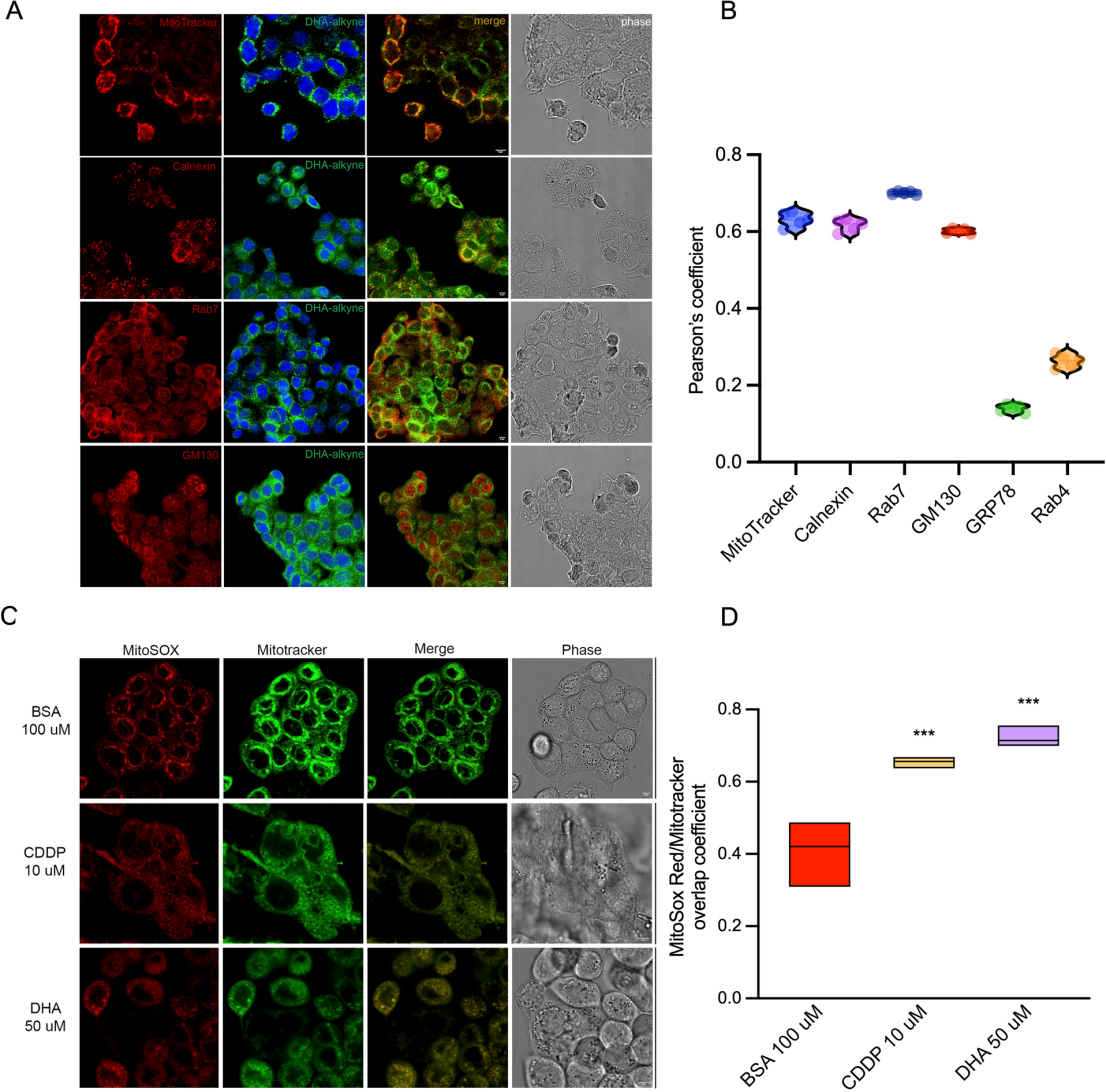
Exogenous DHA localizes in endoplasmic reticulum and induce mitochondrial stress. **A** HT29 cells were seeded, treated with 10 μM DHA-Alkyne for 6 h, then fixed and stained with intracellular compartment-specific markers, including MitoTracker Deep Red FM, Calnexin, Rab7, GM130, GRP78, Rab4 and DAPI. Representative images of single structural marker Mitotracker, Calnexin, Rab7 and GM130 (red), DHA Alkyne and DAPI (green and blue), merge and phase sections are reported. Scale bar 5 μm. **B** Violin boxes recall Pearson’s correlation between DHA-Alkyne and markers previously listed. A Pearson’s correlation value > 0.6 (positive correlation) was reached with Mitotracker, Calnexin, Rab7 and GM130. Confocal images of GRP78 and Rab4 are reported in Supplementary Fig. S3B. **C** HT29 cells were seeded, treated for 6 h with BSA, 10 μM Cisplatin (CDDP) and 50 μM DHA, respectively, then incubated with MitoSox Red and MitoTracker Deep Red FM (LUT adapted in green). Representative pictures of MitoSox, Mitotracker, merge and phase sections are reported for each treatment. **D** The correlation between MitoSox and Mitotracker staining was reported as Overlap coefficient in all tested treatments. Cisplatin was used as control of mitochondrial Reactive Oxygen Species (ROS) induction [49]. Statistical analyses are calculated using BSA as control and plotted as mean ± SEM; ***p < 0.001.

The specific mechanism by which exogenous DHA may promote the ferroptotic process is not yet fully elucidated. A recent study showed that treatment of cancer cells with phospholipids containing DHA in the sn-2 position induces ferroptosis through a dual mechanism involving mitochondrial ROS production and lipid peroxidation at the endoplasmic reticulum (ER) membrane [25]. To investigate whether and where DHA was incorporated within the cells, we employed an alkylated form of DHA that allows its fluorescent visualization using the Click-iT chemistry. DHA was actively taken up by cells and was detectable mainly within intracellular compartments (Fig. 3A). To further investigate the distribution of DHA in different subcellular compartments, cells were co-stained with Calnexin (ER marker), GRP78 (ER stress marker), GM130 (Golgi Apparatus), Mitotracker (mitochondria) and two markers of vesicular trafficking, including Rab7 (late endosome) and Rab4 (early endosome). We observed a prominent accumulation of DHA in the ER, Golgi and late endosomal compartments but no accumulation in the early endosomal compartments and stressed ER (Fig. 3A, B and Supplementary Fig. S3B). Moreover, we observed a considerable DHA localization on mitochondria (Fig. 3A, B). The specificity of DHA labeling was confirmed by negative click-it signal in cells treated with vehicle alone (Supplementary Fig. S3A). By closely examining the mitochondria of DHA-treated cells, we observed morphological alterations resembling those induced by a standard chemotherapeutic agent such as cisplatin (CDDP) (Fig. 3C). To determine whether DHA could affect mitochondrial ROS production, we stained the cells with MitoSOX. DHA-treated cells displayed levels of ROS production—assessed as the ratio between MitoSOX and MitoTracker signals—higher than those observed on untreated cells and further increased when compared to cisplatin treatment (Fig. 3C, D) [25]. These results support the hypothesis that DHA localizes on mitochondria membrane perturbing the electrons transport chain, as previously shown with DHA-containing phosphatidylcholines [26].

### Patient-derived tumor organoids of CRC are extremely sensitive to DHA

**Fig. 4:**
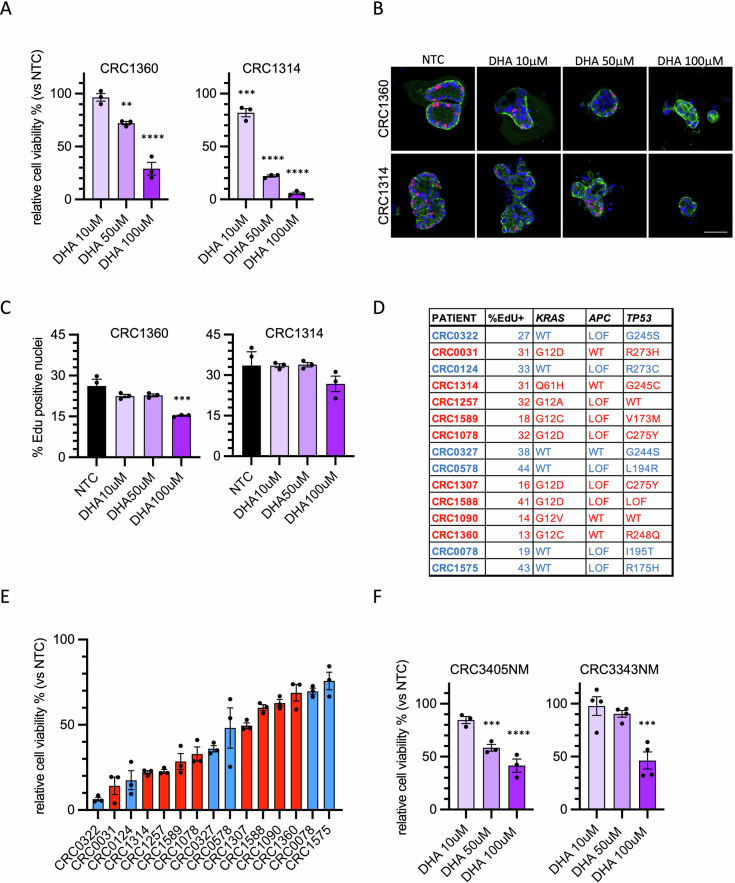
Patient-derived tumor organoids of CRC are extremely sensitive to DHA. **A** PDTOs CRC1314 and CRC1360 were treated three times at 48-h interval with DHA (10 μM, 50 μM and 100 μM) or BSA as control; viability was measured as ATP content after a total of 7 days. The percentage of viable cells in different conditions compared to BSA-treated control (NTC) is plotted as mean ± SEM; **p < 0.01, ***p < 0.001, ****p < 0.0001 *versus* the respective NTC. **B** PDTOs CRC1314 and CRC1360 were treated twice at 48-h interval with DHA (10 μM, 50 μM and 100 μM) or BSA as control; EdU was added to cells for the last 6 h of treatment. Cells were fixed and stained after a total of 5 days. Representative images are shown; EdU is in magenta, E-Cadherin in green, NucBlue™ in blue; scale bar 50 μm. **C** Quantification of the percentage of EdU-positive nuclei (mean ± SEM) in PDTOs CRC1314 and CRC1360 shown in (**B**); ***p < 0.001 *versus* the correspondent NTC. **D** Features of PDTOs used in this study: the first column shows the basal proliferation rate, expressed as the percentage of EdU-positive cells; the second, third and fourth columns show the mutations present in *KRAS*, *APC* and *TP53* genes, respectively. *KRAS*-mutated PDTOs are highlighted in red. **E** All PDTOs were treated three times at 48-h interval with DHA at 50 μM or BSA as control; viability was measured as ATP content after a total of 7 days. The percentage of viable cells compared to BSA-treated control (NTC) is plotted as mean ± SEM. *KRAS*-mutated PDTOs are highlighted in red. **F** Normal human intestinal organoid cultures (CRC3343NM and CRC3405NM) were treated three times at 48-h interval with DHA (10 μM, 50 μM and 100 μM) or BSA as control; viability was measured as ATP content after a total of 7 days. The percentage of viable cells in different conditions compared to BSA-treated control (NTC) is plotted as mean ± SEM; ***p < 0.001, ****p < 0.0001 *versus* the respective NTC.

To evaluate whether the effect of DHA on colorectal cancer cell viability is limited to cell lines or instead affects cell growth of tumor with different mutational alterations, we took advantage of a panel of PDTOs derived from CRC liver metastasis. We initially evaluated the response of two CRC PDTOs, carrying two different KRAS mutations, to various concentrations of DHA. In these preliminary experiments, we observed that even at 10 µM DHA, a significant 15% of growth reduction was achieved in the PDTO CRC1314 (Fig. 4A). At the concentration of 50 µM, both PDTOs showed reduced viability, with a more pronounced effect for the PDTO CRC1314 compared to CRC1360 (Fig. 4A). PDTOs treated with 50 μM of DHA for 5 days showed a substantial size reduction while proliferating cells were still present (Fig. 4B, C). These results confirm the cytotoxic effect of DHA even on PDTOs. Then, we treated a larger panel of PDTOs characterized by different mutational status, with a prevalence of KRAS-mutated tumors (Fig. 4D). KRAS mutations over-activate the MAPK pathway, supporting several cell metabolic alterations, including high oxidative stress [27].

PDTOs were treated with three different concentrations of DHA for seven days, and the results indicate a generally higher sensitivity compared to HT29 cells (Supplementary Fig. S4A). To compare the response across different PDTOs, cell viability at 50 μM DHA was plotted (Fig. 4E). As shown, the range of sensitivity is quite broad, with post-treatment viability levels ranging from 10% to 70% relative to the control. To investigate whether replicative capacity or the mutational status of *KRAS* or *TP53* were involved, we compared these molecular characteristics of the PDTOs with their response to DHA. As shown, the presence of *KRAS* mutations does not appear to significantly influence DHA sensitivity (Fig. 4D, E). Similarly, the presence or absence of inactivating *TP53* mutations is not associated with a greater or lesser response to DHA (Fig. 4D, E). A factor that frequently determines increased sensitivity to certain cytotoxic drugs is the replication rate. However, this parameter, measured as percentage of EdU-incorporating cells, does not appear to be correlated with viability in the presence of DHA (Fig. 4D and Supplementary Fig. S4B).

Finally, taking advantage of the availability in our collection of organoids derived from normal colon tissue, we assessed whether these PDOs were equally sensitive to DHA. The tested PDOs from healthy tissue exhibited a modest decrease of viability following DHA treatment (Fig. 4F). The range of sensitivity to DHA in healthy PDOs was similar to that of less responsive PDTOs, suggesting that specific metabolic alterations of cancer cells could make them more susceptible to DHA-induced stress.

### DHA treatment drives ferroptosis in CRC PDTOs

**Fig. 5:**
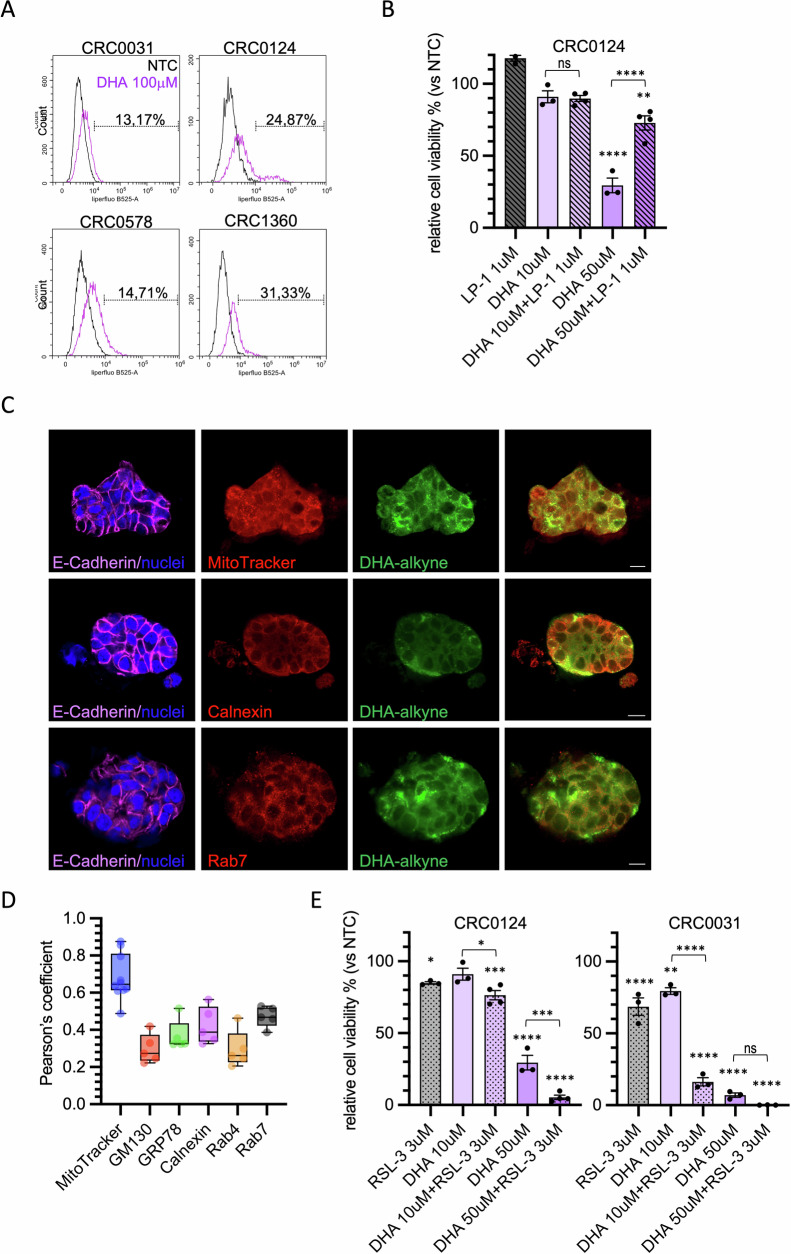
DHA treatment drives ferroptosis in CRC PDTOs. **A** PDTOs CRC0031, CRC0124, CRC0578 and CRC1360 were treated twice at 48-h interval with DHA (100 μM), or BSA as negative control; lipid peroxidation was detected after a total of 72 h using Liperfluo and analized by flow cytometry. Representative histograms are shown; the percentage of cells with fluorescence intensity above the threshold of 10^4^ after treatment with DHA, from 3 independent experiments, is shown on the histograms. **B** CRC0124 was treated three times at 48-h interval with DHA (10 μM and 50 μM) in combination or not with Liproxstatin-1 (LP-1, 1 μM), BSA was used as control; viability was measured as ATP content after a total of 7 days. The percentage of viable cells in different conditions compared to BSA-treated control (NTC) is plotted as mean ± SEM. Statistical significance: DHA 50 μM *versus* NTC ****p < 0.0001; DHA 50 μM + Liproxstatin-1 *versus* NTC **p < 0.01; DHA 10 μM + Liproxstatin-1 *versus* DHA 10 μM ns; DHA 50 μM + Liproxstatin-1 *versus* DHA 50 μM ****p < 0.0001. **C** Representative pictures of CRC0124 stained with DHA alkyne (in green), MitoTracker™, Calnexin and Rab7 (in red), E-cadherin (in magenta) and NucBlue™ (in blue); scale bar 10 μm. **D** Co-localization between DHA and each organelle marker is represented by Pearson’s coefficient plotted in the box and whiskers graph. **E** CRC0124 and CRC0031 were treated three times at 48-h interval with DHA (10 μM and 50 μM) in combination or not with RSL-3 (3 μM), BSA was used as control; viability was measured as ATP content after a total of 7 days. The percentage of viable cells in different conditions compared to BSA-treated control (NTC) is plotted as mean ± SEM. Statistical significance: CRC0124: RSL-3 *versus* NTC *p < 0.05; DHA 10 μM + RSL-3 *versus* NTC ***p < 0,001; DHA 50 μM and DHA 50 μM + RSL-3 *versus* NTC ****p < 0.0001; DHA 10 μM + RSL-3 *versus* DHA 10 mM *p < 0.05; DHA 50 μM + RSL-3 *versus* DHA 50 μM ***p < 0.001; CRC0031: RSL-3, DHA 10 μM + RSL-3, DHA 50 μM and DHA 50 μM + RSL-3 *versus* NTC ****p < 0.0001; DHA 10 μM *versus* NTC **p < 0.01; DHA 10 μM + RSL-3 *versus* DHA 10 μM ****p < 0.0001; DHA 50 μM + RSL-3 *versus* DHA 50 μM ns.

To assess whether the effect of DHA on PDTOs was also due to increased ferroptosis-mediated cell death, and consequently associated with elevated lipid peroxidation, we analyzed the PDTOs using Liperfluo staining followed by flow cytometry analysis. DHA treatment led to an increase in lipid peroxidation in all PTDOs analyzed (Fig. 5A). The involvement of ferroptosis was further supported by the partial rescue of cell viability upon treatment with Liproxstatin-1, a potent radical-trapping antioxidant that inhibits lipid peroxidation–driven ferroptotic cell death, particularly effective at the 50 µM of DHA (Fig. 5B) [28]. We also investigated whether the DHA was internalized in PDTOs. We observed that labeled-DHA was specifically localized to the mitochondria, ER and late endosomes, while it was not detected in the Golgi, early endosomes or plasma membrane (Fig. 5C, D and S5), partially confirming results obtained in HT29 cells.

One of the major limitations of ferroptosis inducers is their high toxicity at effective concentrations, which restricts their clinical applicability. For this reason, we tested whether combining DHA with low doses of the ferroptosis inducer RSL-3, a direct inhibitor of GPX4, could enhance its efficacy [3]. We selected two PDTO models with different sensitivities to DHA and assessed cell viability following combined treatment. CRC0031 showed a strong response to treatment with 10 µM DHA and 3 µM RSL-3. When combined with 50 µM DHA, the efficacy of RSL-3 in reducing cell viability nearly reached 100% (Fig. 5E). In CRC0124, which was less sensitive to DHA, similar effects were observed, but exclusively at the 50 µM DHA concentration (Fig. 5E). These results supported the conclusion that DHA contributes to colorectal cancer cell death by inducing ferroptosis, even in a physiologically relevant model such as PDTOs.

### DHA inhibits the growth of oxaliplatin-tolerant cells in PDTOs

**Fig. 6:**
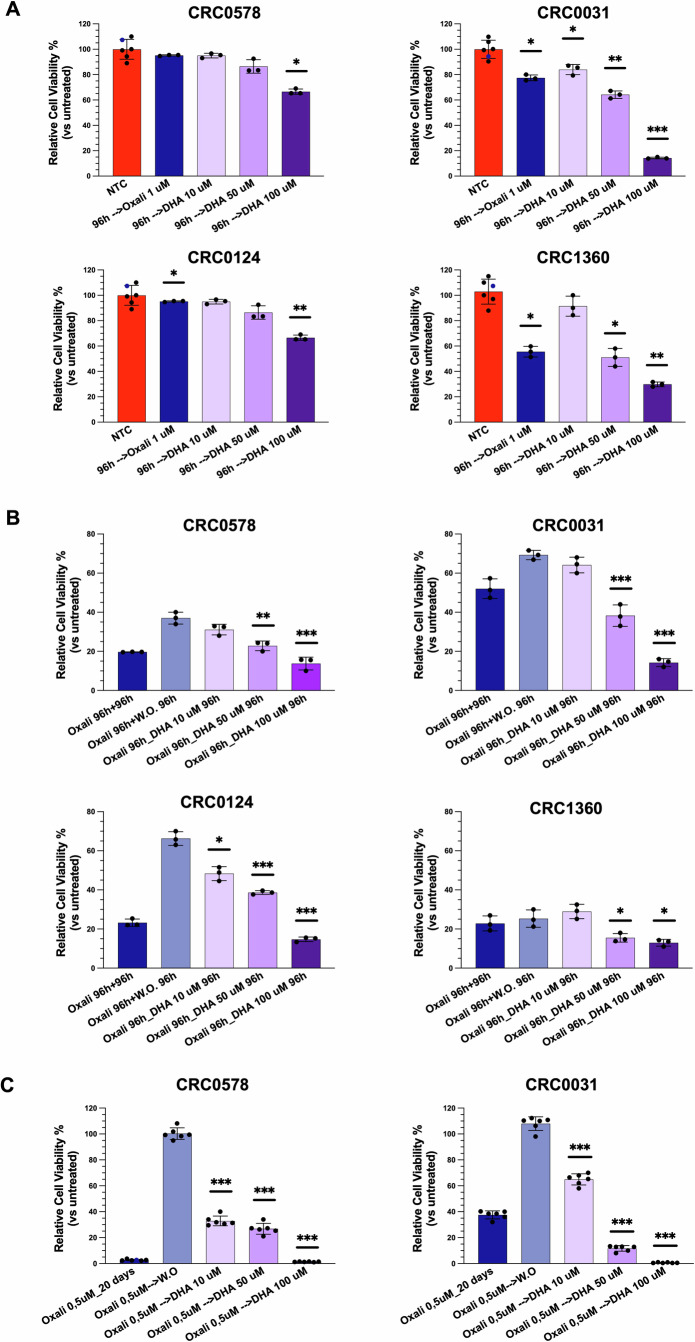
DHA inhibits the growth of oxaliplatin-tolerant cells in PDTOs. PDTOs CRC0578, CRC0031, CRC0124 and CRC1360 were treated with three different treatment schedules: regression trial (**A**) combined sequential trial (**B**) persister cells trial (**C**). **A** PDTOs were digested at single cell, left growing for 6 days, then treated twice at 48-h interval with 1 μM Oxaliplatin (Oxali), 10/50/100 μM DHA and BSA as control. Histograms from three independent experiments are shown and the percentage of viable cells in different conditions compared to the BSA-treated control (NTC) is plotted as mean ± SEM; *p < 0.05, **p < 0.01, ***p < 0.001 *versus* NTC. **B** PDTOs were digested at single cells and left growing for 48 h. The administration of 1 μM Oxaliplatin (twice, at 48-h intervals), was followed by additional 96 h (twice, at 48-h intervals) treatment with either 1 μM Oxaliplatin, Oxaliplatin washout, 10/50/100 μM DHA. Histograms from three independent experiments are shown and the percentage of viable cells in different conditions compared to the BSA-treated control (NTC) is plotted as mean ± SEM; *p < 0.05, **p < 0.01, ***p < 0.001 *versus* Oxaliplatin washout condition. **C** PDTOs CRC0578 and CRC0031 were digested at single cell and left growing for 72 h. The administration of 0.5 μM Oxaliplatin (Oxali) for 10 days (three times, at 72-h intervals), was followed by additional 10 days (three times, at 72-h intervals) treatment with either 0.5 μM Oxaliplatin, Oxaliplatin washout, 10/50/100 μM DHA. Histograms from 6 independent experiments are shown and the percentage of viable cells in different conditions compared to the BSA-treated control (NTC) is plotted as mean ± SEM; *p < 0.05, **p < 0.01, ***p < 0.001 *versus* Oxaliplatin washout condition.

Emerging evidence suggests that ferroptosis represents a vulnerability of DTC cells [29, 30]. The cytotoxic effects of DHA in tumor cells may be leveraged synergistically with antiproliferative chemotherapies acting through mechanisms distinct from ferroptosis.To investigate whether the reduction in cell viability induced by DHA could synergize with a conventional chemotherapeutic treatment for CRC, we compared different schedule treatments of DHA and oxaliplatin on PDTOs. The oxaliplatin was administered after 6 days of PDTO growth, using a regression trial model to evaluate PDTO viability reduction. In this model, oxaliplatin exhibited limited efficacy, achieving at best a ~35% reduction in viability after 96 h of treatment (Fig. 6A). Treatment with DHA at 50 μM resulted in a reduction in cell viability comparable to that induced by oxaliplatin, with the exception of the PDTO CRC0124. At the concentration of 100 µM, DHA led to a markedly higher growth reduction relative to oxaliplatin (Fig. 6A).

These findings prompted us to explore whether a sequential treatment (chemotherapy followed by DHA) could result in superior outcomes compared to chemotherapy alone. In a progression trial model, oxaliplatin effectively halted PDTO growth; however, a rapid regrowth was observed following drug withdrawal, in the majority of cases. Notably, the subsequent administration of DHA prevented PDTO regrowth and, in some cases, further enhanced oxaliplatin efficacy (Fig. 6B). In all PDTOs tested, cell viability at DHA 50 µM was significantly reduced compared to oxaliplatin withdrawal. These results support the hypothesis that DHA may be effective against cell populations that are tolerant or persistent following oxaliplatin treatment. To test this, PDTOs were treated with oxaliplatin for 10 days, after which they ceased to grow, and additional oxaliplatin treatment did not further reduce cell numbers. However, when oxaliplatin was withdrawn, PDTOs resumed growth, proving the presence of tolerant cells. In contrast, DHA treatment markedly impaired this regrowth. Even at the lowest concentration of 10 µM, DHA already exerted a significant effect on organoid growth, whereas at the higher concentration (100 µM) the response exceeded that achieved with chemotherapy treatment alone, leading to near-complete elimination of the PDTOs (Fig. 6C).

Overall, these findings demonstrate that DHA’s ability to induce ferroptosis can be harnessed to potentiate the therapeutic effects of conventional chemotherapy.

## Discussion

In this study, we demonstrate that DHA, a long-chain n-3 PUFA, induces potent cell death through a ferroptosis-dependent mechanism in CRC-derived tumor cells and PDTOs. DHA supplementation reduced cell growth in two-dimensional cultures, with only minimal effects on cellular proliferation. A similar, albeit weaker, effect was observed with EPA and OA, whereas saturated fatty acids (PA) had no detectable impact on cell growth. Strikingly, DHA exhibited markedly enhanced efficacy in CRC metastasis–derived PDTOs. This heightened sensitivity may partly reflect differences in lipid availability in organoid culture conditions, where serum-derived lipids are replaced by defined supplements. However, the reduced response observed in organoids derived from healthy intestinal tissue strongly supports a tumor-specific vulnerability to DHA-induced stress.

In this context, PDTOs represent a particularly informative model, as they preserve tumor-specific metabolic features and three-dimensional architecture that are largely absent in conventional monolayer cultures. Consistent with this interpretation, recent studies have shown that n-3 PUFAs preferentially induce cell death in acid-adapted cancer cells and within the acidic microenvironment of tumor spheroids, where enhanced fatty acid uptake promotes PUFA accumulation and tumor-selective cytotoxicity [26]. Together, these findings suggest that both tumor-associated metabolic reprogramming and three-dimensional tissue organization critically influence PUFA uptake and toxicity.

Mechanistically, DHA-induced cell death became evident after approximately 72 h of exposure at concentrations exceeding 50 µM and was not associated with apoptosis, but rather with ferroptosis, as demonstrated by lipid peroxide accumulation and rescue by ferroptosis inhibitors [31, 32]. Oxidation of PUFA-containing membrane phospholipids is a central trigger of ferroptosis, and although PUFA incorporation alone is insufficient to initiate this process, it strongly modulates its execution [4, 5]. In line with recent work showing that phosphatidylcholines containing DHA at the sn-2 position (PC-DHA) potently induce ferroptosis, we demonstrate that exogenous DHA is actively incorporated into cellular membranes, particularly within the endoplasmic reticulum and late endosomes [25]. This subcellular distribution closely mirrors that reported for PC-DHA and supports the concept that directed remodeling of membrane phospholipid pools underlies ferroptosis sensitivity [25].

Emerging evidence further indicates that extracellular lipid limitation enhances cancer cell sensitivity to ferroptosis, revealing that lipid deprivation activates a PUFA trafficking pathway [33]. These findings indicate that continuous lipid remodeling, regulated in part by environmental conditions, and the directed incorporation of highly unsaturated PUFAs into specific phospholipid pools play a key role in determining ferroptosis sensitivity in cancer cells [33]. Together with observations that dietary n-6 PUFAs induce lineage-specific ferroptosis in *C. elegans*, these data underscore how ferroptosis sensitivity is shaped by metabolic context and cellular differentiation state [34].

DHA accumulation in the endoplasmic reticulum was accompanied by mitochondrial dysfunction and increased ROS production, likely amplifying lipid peroxidation [25]. This effect is particularly pronounced in cancer cells, which already operate under elevated oxidative stress, providing a plausible explanation for the selective sensitivity of CRC PDTOs compared with normal intestinal organoids. Although tumor cells often rely on aerobic glycolysis, their pronounced ferroptotic response to DHA suggests that mitochondrial perturbation remains a critical vulnerability [1, 35]. Notably, drug-tolerant and stem-like cancer cell populations are more dependent on oxidative phosphorylation, supporting the idea that ferroptosis induction may preferentially target cells resistant to conventional cytotoxic or anti-proliferative therapies [35–38].

To date, a major limitation of ferroptosis-inducing drugs has been their severe toxicity, which has precluded their advancement to clinical trials and the assessment of therapeutic combinations involving ferroptosis inducers [35]. Exploiting the properties of DHA, or its analogue PC-DHA—both considered nutritional supplements—in triggering ferroptosis in tumor cells could overcome this limitation. Moreover, it has also been reported that DHA may enhance the effect of ferroptotic drugs, suggesting that a combined administration could represent an effective strategy to induce ferroptosis in cancer cells while reducing toxic effects on normal tissues [39].

Although in vivo validation will be essential to define bioavailability, tissue distribution, and therapeutic windows, the robust and heterogeneous responses observed across CRC PDTOs argue that ferroptosis induction by DHA reflects a broadly conserved tumor vulnerability. Importantly, PDTOs enable direct functional assessment of this vulnerability in a patient-specific manner, strengthening the translational relevance of our findings.

This is particularly evident in sequential treatment models combining DHA with oxaliplatin. In PDTOs, oxaliplatin alone produced limited tumor regression and allowed rapid regrowth upon drug withdrawal, consistent with the persistence of DTC. In contrast, subsequent DHA treatment markedly impaired regrowth and, in some cases, exceeded the cytotoxic effect of chemotherapy alone. The PDTO model is well suited for the study of DTCs, whether they emerge stochastically or originate from stem-like cells, as it preserves tumor phenotypic heterogeneity [40]. Given recent evidence that DTC contribute to minimal residual disease and can be selectively eliminated by ferroptosis induction, our data suggest that DHA may represent a feasible strategy to target this clinically relevant cell population [29, 41].

In general, the possibility of administering a DHA-enriched diet to CRC patients during adjuvant therapy or in the post-treatment setting may enhance the efficacy of chemotherapy without adding significant adverse effects. Moreover, dietary DHA supplementation has been shown to reduce intestinal inflammation induced by chemotherapy as well as by pro-inflammatory dietary regimens, thereby potentially promoting a beneficial effect during anti-tumor therapies [42, 43].

Although the concentrations of DHA used in vitro are quite elevated, the plasmatic levels of free DHA, measured during a clinical trial with dietary administration, are equivalent or higher [44]. Moreover, they are compatible with tissue accumulation achieved through sustained dietary supplementation [7, 45]. Given that DHA is preferentially incorporated into membrane phospholipids, its local enrichment within tumor cells—rather than systemic plasma levels—may be sufficient to lower the ferroptotic threshold.

In the future, it will also be interesting to explore, using PDTO–bacteria co-culture models, how DHA administration may modulate the intestinal microbiota and consequently influence pathological conditions, including polyp formation and colorectal cancer development [46, 47]

Together, these findings provide a strong rationale for exploring dietary or pharmacological DHA-based interventions as adjuvant strategies following chemotherapy and support the use of PDTOs as a predictive platform to guide ferroptosis-based therapeutic approaches in colorectal cancer [12].

## Materials and methods

### Cell lines cultures

HT29 cells were purchased from American Type Culture Collection (LGC Standards Srl) and cultured following ATCC recommended protocols in Dulbecco’s modified Eagle’s medium (DMEM) with high glucose (Gibco), supplemented with 10% fetal bovine serum (FBS, Euroclone), penicillin-streptomycin (Euroclone) and 2 mM L-glutamine (Euroclone). Mycoplasma testing was routinely performed to ensure cell culture quality by the internal facility, and cells were maintained for no more than 20 passages.

L-Wnt-3 A and 293 T-HA-RspoI Fc cells were kindly provided by prof. Trusolino’s Lab. L-Wnt-3 A cells were cultured for 2–3 passages with DMEM with high glucose, plus 10% fetal bovine serum, penicillin-streptomycin and 2 mM L-glutamine and supplemented with Neomycin (0,4 mg/ml). To collect conditioned medium, cells were seeded in an appropriate number of 145-cm^2^ dishes in 20 ml of complete DMEM medium without neomycin. After 7 days, the medium was harvested and centrifuged; the resulting supernatant was then passed through a 0.22-µm Stericup-GP filter.

293 T-HA-RspoI Fc cells were cultured for 2–3 passages with DMEM with high glucose, plus 10% fetal bovine serum, penicillin-streptomycin and 2 mM L-glutamine and supplemented with Zeocin (300 µg/ml). To collect conditioned medium, cells were seeded in an appropriate number of 145-cm^2^ dishes in 20 ml of serum-free Advanced DMEM/F12 (Gibco), plus penicillin-streptomycin and 2 mM L-glutamine. After 10 days, the medium was harvested and centrifuged; the resulting supernatant was then passed through a 0.22-µm Stericup-GP filter. Freshly prepared Wnt3A-CM and RspoI-CM can be stored at −80 °C for long periods of time (>6 months).

### Patient-derived tumor and intestine organoids cultures

Patient-derived tumor organoids (PDTOs) derived from CRC liver metastases were obtained from the Xenturion biobank in our institution (PROFILING protocol No. 001-IRCC-00IIS-10, version 11.0, updated July 13, 2022) [23]. All patients provided informed consent, including for the collection of sex and age information. PDTOs were maintained in Cultrex Basement Membrane Extract (BME Type II, R&D Systems) onto 12-well plates (Corning). Complete medium composition was the following: Dulbecco’s modified Eagle medium/F12 supplemented with penicillin-streptomycin, 2 mM L-glutamine, 1 mM n-Acetyl Cysteine, B27 (Thermo Fisher Scientific), N2 (Thermo Fisher Scientific), and 5 ng/ml EGF (Sigma-Aldrich). PDTOs were routinely tested for Mycoplasma and maintained at 37 °C in a humidified atmosphere of 5% CO_2_.

Human intestinal organoid cultures were established from fresh biopsies of healthy small intestine or colon. All patients signed a dedicated informed consent in accordance with guidelines of the ALFAOMEGA Master Observational Trial (NCT04120935) [48]. The study protocol was sponsored by IFOM ETS - The AIRC Institute of Molecular Oncology and approved by the Ethical Committee of each participating center. Tissues were first cut into small fragments and then washed with cold PBS. To extract intestinal crypts, tissue fragments were incubated for 30 minutes at 4 °C with a gentle shaking in 2 mM EDTA cold chelation buffer (5,6 mM Na2HPO4, 8 mM KH2PO4, 96,2 mM NaCl, 1,6 mM KCl, 43,4 mM sucrose, 54,9 mM D-sorbitol, 0,5 mM DTT). After allowing tissue fragments to settle down under normal gravity for 1 minute, EDTA buffer was removed and fragments were vigorously resuspended in cold chelation buffer using 10-ml pipette to isolate intestinal crypts. This procedure of resuspension/sedimentation was repeated at least 4–5 times (supernatant was inspected for the presence of crypts after each passage). The supernatants containing crypts were filtered (100 µm-filter) and collected in 50-ml tube coated with BSA. Intestinal crypts were centrifuged at 300 g for 3 min, washed with cold chelation buffer and centrifuged again at 200 × *g* for 3 min. Intestinal crypts were then seeded in 24-wells plate with complete medium plus 10 µM Y27632 (MedChemExpress) and 3 µM CHIR99021 (MedChemExpress) for the first 5–6 days. Complete medium composition was the following: basal medium (Advanced Dulbecco’s modified Eagle medium/F12, penicillin-streptomycin, 2 mM L-glutamine, 1 mM n-Acetyl Cysteine, 10 mM HEPES, B27), supplemented with 50% Wnt3a CM, 10% RspoI-CM, EGF 50 ng/ml, Noggin (Peprotech) 100 ng/ml, A8301 (MedChemExpress) 500 nM, Gastrin (Merck) 10 nM, Nicotinammide (Sigma-Aldrich) 5 mM, Primocin (InvivoGen), IGF1 (Peprotech) 100 ng/ml, FGF2 (Peprotech) 50 ng/ml.

### Drugs, enzymatic inhibitors and stock solutions of fatty acids

Erastin, Ferrostatin-1, RSL-3, Liproxstatin-1, ABT-263 and Oxaliplatin were from MedChemExpress. Docosahexaenoic Acid (DHA, MedChemExpress), Eicosapentaenoic acid (EPA, MedChemExpress) and Palmitic acid (PA, MedChemExpress) were dissolved in 100% ethanol to a final concentration of 100 mM; Oleic acid (OA, MedChemExpress) was dissolved in 100% ethanol to a final concentration of 10 mM. 1 ml of these solutions were mixed with 9 ml of 20% fatty acid-free BSA in phosphate-buffered saline (PBS) at 50 °C for 1 h, yielding a final stock solution of 10 mM for DHA, EPA, PA, and of 1 mM for OA. A control BSA solution was prepared by mixing 1 ml of 100% ethanol with 9 ml of 20% fatty acid-free BSA in PBS.

### Viability assays

Cell lines viability experiments were performed in 96-well plates, with 500 cells/well. After 2 days from seeding, cells were treated with the modalities indicated in the figure legends. Cell viability was measured by ATP content using the Cell Titer-Glo luminescent assay kit (Promega), according to manufacturer’s instructions. Ratios between treated and untreated cells were calculated.

PDTOs and normal intestinal organoids viability experiments were performed in 96-well plates, coated with a thin layer of BME in each well. PDTOs or normal intestinal organoids were washed with PBS, incubated with TrypLE™ Express solution (Thermo Fisher Scientific) for 5 min at 37 °C and vigorously pipetted to obtain a single cell suspension.

Cells were seeded in complete culture medium supplemented with 2% BME. After 2 days from seeding, PDTOs were treated with the modalities indicated in the figure legends. Cell viability was measured by ATP content using the Cell Titer-Glo luminescent assay kit, according to manufacturer’s instructions. Ratios between treated and untreated cells were calculated.

### Lipid peroxidation detection

#### Liperfluo

Liperfluo (Dojindo) was used according to the manufacturer’s protocol with minimal modifications. HT29 were seeded in 6-well cell culture plates and were treated twice at 48-h interval, then detection of lipid peroxidation was performed after a total of 72 h. Liperfluo was administered for 30 minutes at 37 °C in serum free medium (final concentration 2,5 µmol/l). After incubation, cells were washed twice with Hank’s balanced salt solution (HBSS) (Gibco) and prepared for flow cytometry analysis. For cell imaging, HT29 were plated onto 96-well black cell culture plates (Ibidi) and lipid peroxidation was detected with the protocol described above. HT29 were then observed by an imaging automated system for multiplex in-cell and in-tissue analyses (Nikon LIPSI). PDTOs were seeded in BME-domes and were treated twice at an interval of 48 h, then detection of lipid peroxidation is performed after a total of 72 h. PDTOs were dissociated from the BME matrix by pipetting and Liperfluo was administered for 30 minutes at 37 °C in DMEM/F12 medium (final concentration 2,5 µmol/l). After incubation, PDTOs were washed twice with HBSS and prepared for analysis. HT29 were analysed with Beckman Coulter Cyan ADP, while PDTOs were analysed with Beckman Coulter Cytoflex LX. Gating strategies are shown in figure S6. Cells were initially gated based on forward scatter (FSC) and side scatter (SSC) parameters to exclude debris. Doublets were excluded using Pulse width versus FSC-A or SSC-A versus SSC-H discrimination. Viable single cells (DAPI-negative) were then analyzed for Liperfluo fluorescence intensity. Percentages indicate the proportion of cells retained for analysis after each gating step.

#### Bodipy® 581/591 C11

HT29 were plated onto 96-well black cell culture plates and treated as described above. Image-iT® lipid peroxidation kit (Thermo Fisher Scientific) was used to visualize lipid peroxidation, according to the manufacturer’s protocol. HT29 were then observed by an imaging automated system for multiplex in-cell and in-tissue analyses (Nikon LIPSI). Images were acquired at two separate wavelengths: one at excitation/emission of 581/591 nm for the reduced dye, and the other at excitation/emission of 488/510 nm for the oxidized dye. The ratio of mean fluorescence intensities of the dye at 590 nm and 510 nm was used as the readout for lipid peroxidation.

#### Malondialdehyde (MDA) assay

Lipid peroxidation was measured using the Lipid Peroxidation MDA Assay Kit (Sigma) according to the manufacturer’s instructions. HT29 were seeded in 6-well cell culture plates in duplicate and were treated as described above. After treatment, pellets of cells (max 2 × 10^6^) were homogenized on ice in 300 µL MDA Lysis Buffer containing 3 µL butylated hydroxytoluene (BHT, 100×), then centrifuged at 13,000 × *g* for 15 min; 200 µL of supernatant was used for the subsequent MDA–TBA reaction, by adding 600 µL of thiobarbituric acid (TBA) solution to samples or standard. After incubation at 95 °C for 60 min, 200 µL of each reaction was transferred to a 96-well plate for reading. Absorbance was read at 532 nm; standards were run on each plate and blank values subtracted. MDA amounts (nmol per well) were calculated from the standard curve and values from treated samples were normalized to the NTC.

### Immunofluorescence

Cell proliferation was analyzed by Click-iT™ EdU Alexa Fluor® 647 Imaging kit (Thermo Fisher Scientific). In brief, 10 × 10^3^ HT29 were plated on glass coverslips in 24-well plates in complete medium for 48 h, then they were treated twice at an interval of 48 h with different fatty acids as indicated in figure legend. PDTOs were seeded as single cells (10 × 10^4^/well) on 12-well chambered slides (Ibidi) in 2% BME complete culture medium. After 2 days from seeding, PDTOs were treated with DHA with the time scheduling described for HT29. EdU was added to cells or PDTOs for the last 6 h of treatment. Then cells/PDTOs were fixed and stained following manufacturer’s instructions. Four random fields of each sample from three independent experiments were photographed at the confocal microscope at low magnification and Alexa Fluor® 647 positive nuclei were counted. To measure the proportion of EdU-positive cells, nuclei were counterstained with Dapi.

For the different immunofluorescence analysis, HT29 and PDTOs were plated as illustrated above and treated as described in figure legends. After treatment, cells were fixed with 4% paraformaldehyde in PBS for 10 (HT29) or 20 (PDTOs) min. After fixation, cells were rinsed three times with PBS, quenched with 50 mM NH_4_Cl for 20 min at room temperature, washed twice with PBS, and then permeabilized at room temperature with PBS 0.2% Triton X-100 for 8 min (HT29) or PBS 0.5% Triton X-100 for 20 min (PDTOs). After two washes with PBS, coverslips were blocked for 1 h at room temperature with PBS 1% donkey serum (HT29) or PBS 0.1% Triton X-100, 10% donkey serum (PDTOs), and incubated with primary antibodies overnight at 4° C in a humidified chamber. The following primary antibodies were used: anti-cleaved Caspase 3 (CST 9664), anti-GM130 (CST 124805), anti-Calnexin (Merck HPA00943), anti-GRP78 (CST 3177S), anti-Rab4 (CST 2167), anti-Rab7 (CST 9367) and anti-ECadherin (R&D Systems, AF648). After three washes with PBS, coverslips were incubated for 1 h at room temperature in a humidified chamber with Alexa Fluor® fluorescent secondary antibodies (Thermo Fisher Scientific). Where indicated, MitoTracker™ Deep Red FM and MitoSOX™ Red (Thermo Fisher Scientific) dyes were used following manufacturer’s instructions.

To study the intracellular localization of DHA, HT29 and PDTOs were grown on glass coverslips or 12-well chambered slides as described. Then, cells/PDTOs were incubated with 10 or 50 µM DHA alkyne (Cayman Chemical) for 6 h. After fixation with 4% paraformaldehyde in PBS and permeabilization (0,1% Triton X100 in PBS for 2 minutes for HT29, 0,5% Triton X100 in PBS for 20 min for PDTOs), cells were washed with 3% BSA in PBS. The Click-iT reaction (Cu(I)-catalyzed azide-alkyne cycloaddition) was performed with Click-iT™ Plus Alexa Fluor® Picolyl Azide Toolkit (Thermo Scientific) according to manufacturer’s instructions.

Coverslips were then rinsed three times with PBS, mounted with ProLong™ Glass Antifade mountant with NucBlue™, and analyzed using a confocal microscope (Stellaris 5 WLL NIR, Leica). Confocal images are maximum projections of a *z*-section of approximately 1.50 µm for HT29 or a single slice for PDTOs. The images were arranged and labeled using Fiji software.

Pearson’s colocalization coefficient (r) was used to quantify the overlap between the fluorescence signals of DHA alkyne and each organelle marker. The correlation between Mitosox and Mitotracker signals was quantified as Overlap coefficient Ratio. The analysis was conducted using the JACoP plugin in Fiji.

### Statistical analysis

Sample size was determined empirically based on established practice for these in vitro assays. For both cell-line and PDO experiments, each condition was tested in technical replicates within an experiment and the experiment was independently repeated at least three times. The number of biological (nontechnical) replicates for each experiment is reported in the figure legends, alongside the adopted statistical tests and metrics. Given the exploratory nature of the study and the variability intrinsic to patient-derived models, we did not perform an a priori formal power calculation. Statistical analysis was performed using GraphPad software. Descriptive statistics (means and standard errors) were calculated for each group. One-way analysis of variance (ANOVA) with multiple comparisons was conducted to assess the statistical significance across all experiments, except for the time-course experiment for which two-way ANOVA was utilized. Only statistically significant results are indicated by asterisks in the graphs, with details reported in the figure legends.

## Supplementary information

Supplemental figures

## Data Availability

Requests for further information and resources should be directed to and will be fulfilled by the lead contact Luca Primo (luca.primo@unito.it).

## References

[1] Stockwell BR, Friedmann Angeli JP, Bayir H, Bush AI, Conrad M, Dixon SJ, et al. Ferroptosis: a regulated cell death nexus linking metabolism, redox biology, and disease. Cell. 2017;171:273–85. 28985560 10.1016/j.cell.2017.09.021 PMC5685180

[2] Dixon SJ, Lemberg KM, Lamprecht MR, Skouta R, Zaitsev EM, Gleason CE, et al. Ferroptosis: an iron-dependent form of nonapoptotic cell death. Cell. 2012;149:1060–72. 22632970 10.1016/j.cell.2012.03.042 PMC3367386

[3] Yang WS, SriRamaratnam R, Welsch ME, Shimada K, Skouta R, Viswanathan VS, et al. Regulation of ferroptotic cancer cell death by GPX4. Cell. 2014;156:317–31. 24439385 10.1016/j.cell.2013.12.010 PMC4076414

[4] Yang WS, Kim KJ, Gaschler MM, Patel M, Shchepinov MS, Stockwell BR. Peroxidation of polyunsaturated fatty acids by lipoxygenases drives ferroptosis. Proc Natl Acad Sci USA. 2016;113:E4966–75. 27506793 10.1073/pnas.1603244113 PMC5003261

[5] Doll S, Proneth B, Tyurina YY, Panzilius E, Kobayashi S, Ingold I, et al. ACSL4 dictates ferroptosis sensitivity by shaping cellular lipid composition. Nat Chem Biol. 2017;13:91–98. 27842070 10.1038/nchembio.2239 PMC5610546

[6] Fuentes NR, Mlih M, Barhoumi R, Fan YY, Hardin P, Steele TJ, et al. Long-chain n-3 fatty acids attenuate oncogenic KRas-driven proliferation by altering plasma membrane nanoscale proteolipid composition. Cancer Res. 2018;78:3899–912. 29769200 10.1158/0008-5472.CAN-18-0324 PMC6050089

[7] Calder PC. Marine omega-3 fatty acids and inflammatory processes: effects, mechanisms and clinical relevance. Biochim Biophys Acta. 2015;1851:469–84. 25149823 10.1016/j.bbalip.2014.08.010

[8] Aldoori J, Cockbain AJ, Toogood GJ, Hull MA. Omega-3 polyunsaturated fatty acids: moving towards precision use for prevention and treatment of colorectal cancer. Gut. 2022;71:822–37. 35115314 10.1136/gutjnl-2021-326362

[9] Siegel RL, Miller KD, Fuchs HE, Jemal A. Cancer statistics, 2022. CA Cancer J Clin. 2022;72:7–33. 35020204 10.3322/caac.21708

[10] Fearon ER, Vogelstein B. A genetic model for colorectal tumorigenesis. Cell. 1990;61:759–67. 2188735 10.1016/0092-8674(90)90186-i

[11] Cunningham D, Humblet Y, Siena S, Khayat D, Bleiberg H, Santoro A, et al. Cetuximab monotherapy and cetuximab plus irinotecan in irinotecan-refractory metastatic colorectal cancer. N Engl J Med. 2004;351:337–45. 15269313 10.1056/NEJMoa033025

[12] Pu Y, Li L, Peng H, Liu L, Heymann D, Robert C, et al. Drug-tolerant persister cells in cancer: the cutting edges and future directions. Nat Rev Clin Oncol. 2023;20:799–813. 37749382 10.1038/s41571-023-00815-5

[13] MacLean CH, Newberry SJ, Mojica WA, Khanna P, Issa AM, Suttorp MJ, et al. Effects of omega-3 fatty acids on cancer risk: a systematic review. JAMA. 2006;295:403–15. 16434631 10.1001/jama.295.4.403

[14] Volpato M, Hull MA. Omega-3 polyunsaturated fatty acids as adjuvant therapy of colorectal cancer. Cancer Metastasis Rev. 2018;37:545–55. 29971573 10.1007/s10555-018-9744-y PMC6133177

[15] Sun G, Fuller H, Fenton H, Race AD, Downing A, Rees CJ, et al. The relationship between dietary and supplemental omega-3 highly unsaturated fatty acid intake, blood and tissue omega-3 highly unsaturated fatty acid concentrations, and colorectal polyp recurrence: a secondary analysis of the seAFOod polyp prevention trial. J Nutr. 2025;155:549–58. 39675479 10.1016/j.tjnut.2024.12.004 PMC7617434

[16] Song M, Ou FS, Zemla TJ, Hull MA, Shi Q, Limburg PJ, et al. Marine omega-3 fatty acid intake and survival of stage III colon cancer according to tumor molecular markers in NCCTG Phase III trial N0147 (Alliance). Int J Cancer. 2019;145:380–9. 30623420 10.1002/ijc.32113 PMC6525069

[17] Cockbain AJ, Volpato M, Race AD, Munarini A, Fazio C, Belluzzi A, et al. Anticolorectal cancer activity of the omega-3 polyunsaturated fatty acid eicosapentaenoic acid. Gut. 2014;63:1760–8. 24470281 10.1136/gutjnl-2013-306445

[18] Liu H, Chen J, Shao W, Yan S, Ding S. Efficacy and safety of Omega-3 polyunsaturated fatty acids in adjuvant treatments for colorectal cancer: a meta-analysis of randomized controlled trials. Front Pharmacol. 2023;14:1004465. 37144220 10.3389/fphar.2023.1004465 PMC10151497

[19] Hull MA, Ow PL, Ruddock S, Brend T, Smith AF, Marshall H, et al. Randomised, placebo-controlled, phase 3 trial of the effect of the omega-3 polyunsaturated fatty acid eicosapentaenoic acid (EPA) on colorectal cancer recurrence and survival after surgery for resectable liver metastases: EPA for metastasis trial 2 (EMT2) study protocol. BMJ Open. 2023;13:e077427. 38030258 10.1136/bmjopen-2023-077427 PMC10689403

[20] van de Wetering M, Francies HE, Francis JM, Bounova G, Iorio F, Pronk A, et al. Prospective derivation of a living organoid biobank of colorectal cancer patients. Cell. 2015;161:933–45. 25957691 10.1016/j.cell.2015.03.053 PMC6428276

[21] Sachs N, Clevers H. Organoid cultures for the analysis of cancer phenotypes. Curr Opin Genet Dev. 2014;24:68–73. 24657539 10.1016/j.gde.2013.11.012

[22] Schutgens F, Clevers H. Human organoids: tools for understanding biology and treating diseases. Annu Rev Pathol. 2020;15:211–34. 31550983 10.1146/annurev-pathmechdis-012419-032611

[23] Leto SM, Grassi E, Avolio M, Vurchio V, Cottino F, Ferri M, et al. XENTURION is a population-level multidimensional resource of xenografts and tumoroids from metastatic colorectal cancer patients. Nat Commun. 2024;15:7495. 39209908 10.1038/s41467-024-51909-2 PMC11362617

[24] Jayathilake AG, Luwor RB, Nurgali K, Su XQ. Molecular mechanisms associated with the inhibitory role of long chain n-3 PUFA in colorectal cancer. Integr Cancer Ther. 2024;23:15347354241243024. 38708673 10.1177/15347354241243024 PMC11072084

[25] Qiu B, Zandkarimi F, Bezjian CT, Reznik E, Soni RK, Gu W, et al. Phospholipids with two polyunsaturated fatty acyl tails promote ferroptosis. Cell. 2024;187:1177–90.e1118. 38366593 10.1016/j.cell.2024.01.030 PMC10940216

[26] Dierge E, Debock E, Guilbaud C, Corbet C, Mignolet E, Mignard L, et al. Peroxidation of n-3 and n-6 polyunsaturated fatty acids in the acidic tumor environment leads to ferroptosis-mediated anticancer effects. Cell Metab. 2021;33:1701–15.e1705. 34118189 10.1016/j.cmet.2021.05.016

[27] Kerk SA, Papagiannakopoulos T, Shah YM, Lyssiotis CA. Metabolic networks in mutant KRAS-driven tumours: tissue specificities and the microenvironment. Nat Rev Cancer. 2021;21:510–25. 34244683 10.1038/s41568-021-00375-9 PMC10257891

[28] Friedmann Angeli JP, Schneider M, Proneth B, Tyurina YY, Tyurin VA, Hammond VJ, et al. Inactivation of the ferroptosis regulator Gpx4 triggers acute renal failure in mice. Nat Cell Biol. 2014;16:1180–91. 25402683 10.1038/ncb3064 PMC4894846

[29] Rodriguez R, Schreiber SL, Conrad M. Persister cancer cells: Iron addiction and vulnerability to ferroptosis. Mol Cell. 2022;82:728–40. 34965379 10.1016/j.molcel.2021.12.001 PMC9152905

[30] Hangauer MJ, Viswanathan VS, Ryan MJ, Bole D, Eaton JK, Matov A, et al. Drug-tolerant persister cancer cells are vulnerable to GPX4 inhibition. Nature. 2017;551:247–50. 29088702 10.1038/nature24297 PMC5933935

[31] Jiang X, Stockwell BR, Conrad M. Ferroptosis: mechanisms, biology and role in disease. Nat Rev Mol Cell Biol. 2021;22:266–82. 33495651 10.1038/s41580-020-00324-8 PMC8142022

[32] Murray MB, Leak LB, Lee WC, Dixon SJ. Protocol for detection of ferroptosis in cultured cells. STAR Protoc. 2023;4:102457. 37556320 10.1016/j.xpro.2023.102457 PMC10432795

[33] Sokol KH, Lee CJ, Rogers TJ, Waldhart A, Ellis AE, Madireddy S, et al. Lipid availability influences ferroptosis sensitivity in cancer cells by regulating polyunsaturated fatty acid trafficking. Cell Chem Biol. 2025;32:408–22.e406. 39442523 10.1016/j.chembiol.2024.09.008 PMC11928283

[34] Perez MA, Magtanong L, Dixon SJ, Watts JL. Dietary lipids induce ferroptosis in *Caenorhabditis elegans* and human cancer cells. Dev Cell. 2020;54:447–54.e444. 32652074 10.1016/j.devcel.2020.06.019 PMC7483868

[35] Ubellacker JM, Dixon SJ. Prospects for ferroptosis therapies in cancer. Nat Cancer. 2025;6:1326–36. 10.1038/s43018-025-01037-7 PMC13066655 40826242

[36] Viale A, Pettazzoni P, Lyssiotis CA, Ying H, Sánchez N, Marchesini M, et al. Oncogene ablation-resistant pancreatic cancer cells depend on mitochondrial function. Nature. 2014;514:628–32. 25119024 10.1038/nature13611 PMC4376130

[37] Keoh LQ, Chiu CF, Ramasamy TS. Metabolic plasticity and cancer stem cell metabolism: exploring the glycolysis-OXPHOS switch as a mechanism for resistance and tumorigenesis. Stem Cell Rev Rep. 2025;21:2446–68. 40880049 10.1007/s12015-025-10956-y PMC12504402

[38] Elgendy SM, Alyammahi SK, Alhamad DW, Abdin SM, Omar HA. Ferroptosis: an emerging approach for targeting cancer stem cells and drug resistance. Crit Rev Oncol Hematol. 2020;155:103095. 32927333 10.1016/j.critrevonc.2020.103095

[39] Shan K, Feng N, Zhu D, Qu H, Fu G, Li J, et al. Free docosahexaenoic acid promotes ferroptotic cell death via lipoxygenase dependent and independent pathways in cancer cells. Eur J Nutr. 2022;61:4059–75. 35804267 10.1007/s00394-022-02940-w

[40] Dhimolea E, de Matos Simoes R, Kansara D, Al’Khafaji A, Bouyssou J, Weng X, et al. An embryonic diapause-like adaptation with suppressed Myc activity enables tumor treatment persistence. Cancer Cell. 2021;39:240–56.e211. 33417832 10.1016/j.ccell.2020.12.002 PMC8670073

[41] Russo M, Chen M, Mariella E, Peng H, Rehman SK, Sancho E, et al. Cancer drug-tolerant persister cells: from biological questions to clinical opportunities. Nat Rev Cancer. 2024;24:694–717. 39223250 10.1038/s41568-024-00737-z PMC12622869

[42] Alcorta A, Lopez-Gomez L, Capasso R, Abalo R. Vitamins and fatty acids against chemotherapy-induced intestinal mucositis. Pharmacol Ther. 2024;261:108689. 38972454 10.1016/j.pharmthera.2024.108689

[43] Sztolsztener K, Harasim-Symbor E, Chabowski A, Konstantynowicz-Nowicka K. Cannabigerol as an anti-inflammatory agent altering the level of arachidonic acid derivatives in the colon tissue of rats subjected to a high-fat high-sucrose diet. Biomed Pharmacother. 2024;178:117286. 39128189 10.1016/j.biopha.2024.117286

[44] Aldoori J, Zulyniak MA, Toogood GJ, Hull MA. Plasma n-3 polyunsaturated fatty acid levels and colorectal cancer risk in the UK Biobank: evidence of nonlinearity, as well as tumor site- and sex-specificity. Cancer Epidemiol Biomarkers Prev. 2025;34:394–404. 39704623 10.1158/1055-9965.EPI-24-1154

[45] Browning LM, Walker CG, Mander AP, West AL, Madden J, Gambell JM, et al. Incorporation of eicosapentaenoic and docosahexaenoic acids into lipid pools when given as supplements providing doses equivalent to typical intakes of oily fish. Am J Clin Nutr. 2012;96:748–758. 22932281 10.3945/ajcn.112.041343 PMC3441107

[46] Agagunduz D, Cocozza E, Cemali O, Bayazit AD, Nani MF, Cerqua I, et al. Understanding the role of the gut microbiome in gastrointestinal cancer: a review. Front Pharmacol. 2023;14:1130562. 36762108 10.3389/fphar.2023.1130562 PMC9903080

[47] Hajjar R, Mars RAT, Kashyap PC. Harnessing the microbiome for cancer therapy. Nat Rev Microbiol. 2026:1–16. 10.1038/s41579-025-01268-6 PMC12914569 41486395

[48] Lazzari L, Corti G, Picco G, Isella C, Montone M, Arcella P, et al. Patient-derived xenografts and matched cell lines identify pharmacogenomic vulnerabilities in colorectal cancer. Clin Cancer Res. 2019;25:6243–59. 31375513 10.1158/1078-0432.CCR-18-3440 PMC7611232

[49] Kleih M, Bopple K, Dong M, Gaissler A, Heine S, Olayioye MA, et al. Direct impact of cisplatin on mitochondria induces ROS production that dictates cell fate of ovarian cancer cells. Cell Death Dis. 2019;10:851. 31699970 10.1038/s41419-019-2081-4 PMC6838053

