# Supplementary material for: Omega-3 fatty acid DHA induces ferroptosis in colorectal cancer patient-derived organoids and drug-tolerant cells
Source: Cell Death Dis. 2026 Apr 11;17(1):464. doi: 10.1038/s41419-026-08744-8 (PMC13180983; doi:10.1038/s41419-026-08744-8)
Supplement: Supplementary file 1 — Supplemental figures [file 41419_2026_8744_MOESM1_ESM.pdf]

SUPPLEMENTAL MATERIAL

This supplemental material contains 6 supplemental figures and relative legends

SUPPLEMENTAL FIGURE LEGENDS

Figure S1. HT29 were treated twice at 48-hour intervals with DHA, EPA, PA and OA (10 μM, 50 μM and 100 μM), or BSA as control (NTC); EdU was added to cells for the last 6 hours of treatment. Cells were fixed and stained after a total of 72 hours. Representative images are shown; EdU is in red, E-Cadherin in green, NucBlue™ in blue; scale bar 20 μm.

Figure S2. A) Representative images of Liperfluo staining (in green) in HT29, after treatment with DHA 50 and 100 μM (same schedule described in figure 2A); scale bar 50 μm. B) Representative images of Bodipy® 581/591 C11 staining in HT29, after treatment with DHA 50 and 100 μM (schedule described in figure 2C); reduced dye is in pink, oxidized dye is in green, scale bar 50 μm. C) HT29 were treated twice at 48-hour interval with DHA (50 μM), Erastin (5 μM), Ferrostatin-1 (10 μM) in combination with DHA or Erastin and BSA as negative control; lipid peroxidation was detected after a total of 72 hours using Liperfluo and analized by flow citometry. Representative histograms are shown; the percentage of cells with fluorescence signal intensity above the threshold of 50, from 3 independent experiments, is plotted as mean±SEM; **p<0,01, ***p<0,001, ****p<0,0001 *versus* NTC; Erastin *versus* Erastin + Ferrostatin-1 ns; DHA 50 μM *versus* DHA 50 μM + Ferrostatin-1 **p<0,01; DHA 100 μM *versus* DHA 100 μM + Ferrostatin-1 ****p<0,0001.

Figure S3. A) Negative control of Click-iT reaction (in green) in HT29; E-Cadherin in red, NucBlue™ in blue; scale bar 10 μm. B) Representative pictures of single structural marker

GRP78 and Rab4 (red), DHA Alkyne and DAPI (green and blue), merge and phase sections are reported. Scale bar 5 mm.

Figure S4. A) All PDTOs were treated three times at 48-hour interval with DHA (10 μM, 50 μM and 100 μM) or BSA as control; viability was measured as ATP content after a total of 7 days. For each PDTO, the percentage of viable cells in different conditions compared to the BSA-treated control (NTC) is plotted as mean±SEM; *$p<0{,}05$, **$p<0{,}01$, ***$p<0{,}001$, ****$p<0{,}0001$ *versus* respective NTC. B) All PDTOs were grown for 3 days in complete medium; EdU was added to the medium for the last 6 hours before fixation and staining. The percentage of EdU-positive nuclei in each condition is plotted as mean±SEM.

Figure S5. A) Representative pictures of CRC0124 stained with DHA alkyne (in green), Rab4, GRP78 and GM130 (in red), Ecadherin (in magenta) and NucBlue™ (in blue); scale bar 10 μm.

Figure S6. Representative flow cytometry images showing the strategy that was implemented for the analysis of lipid peroxidation with Liperfluo in HT29 cells (A, experiments shown in figures 2 and S2) and PDTOs (B, experiments shown in figure 5).

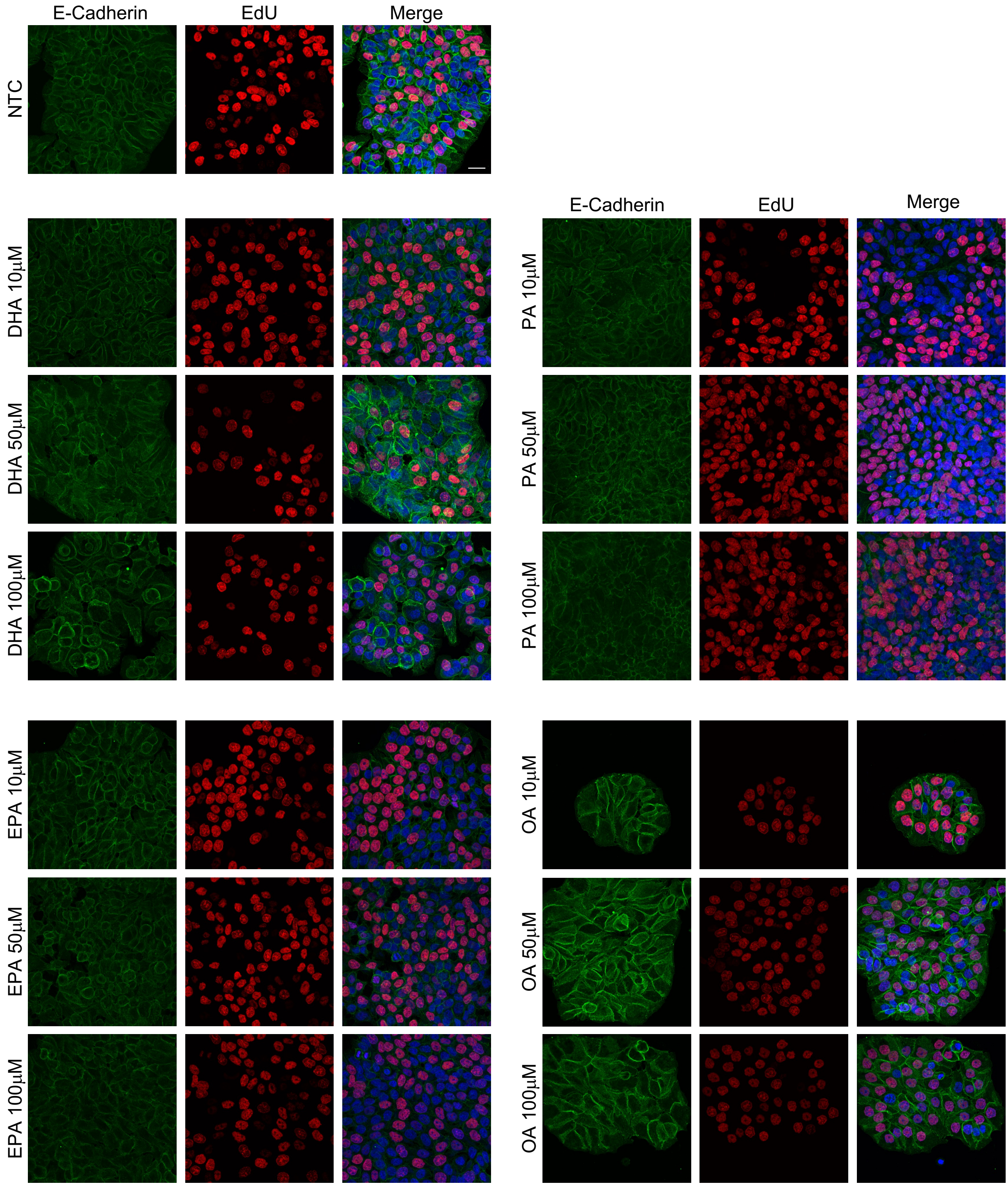
E-Cadherin
EdU
Merge
NTC
DHA 10µM
DHA 50µM
DHA 100µM
EPA 10µM
EPA 50µM
EPA 100µM
PA 10µM
PA 50µM
PA 100µM
OA 10µM
OA 50µM
OA 100µM

Figure S1

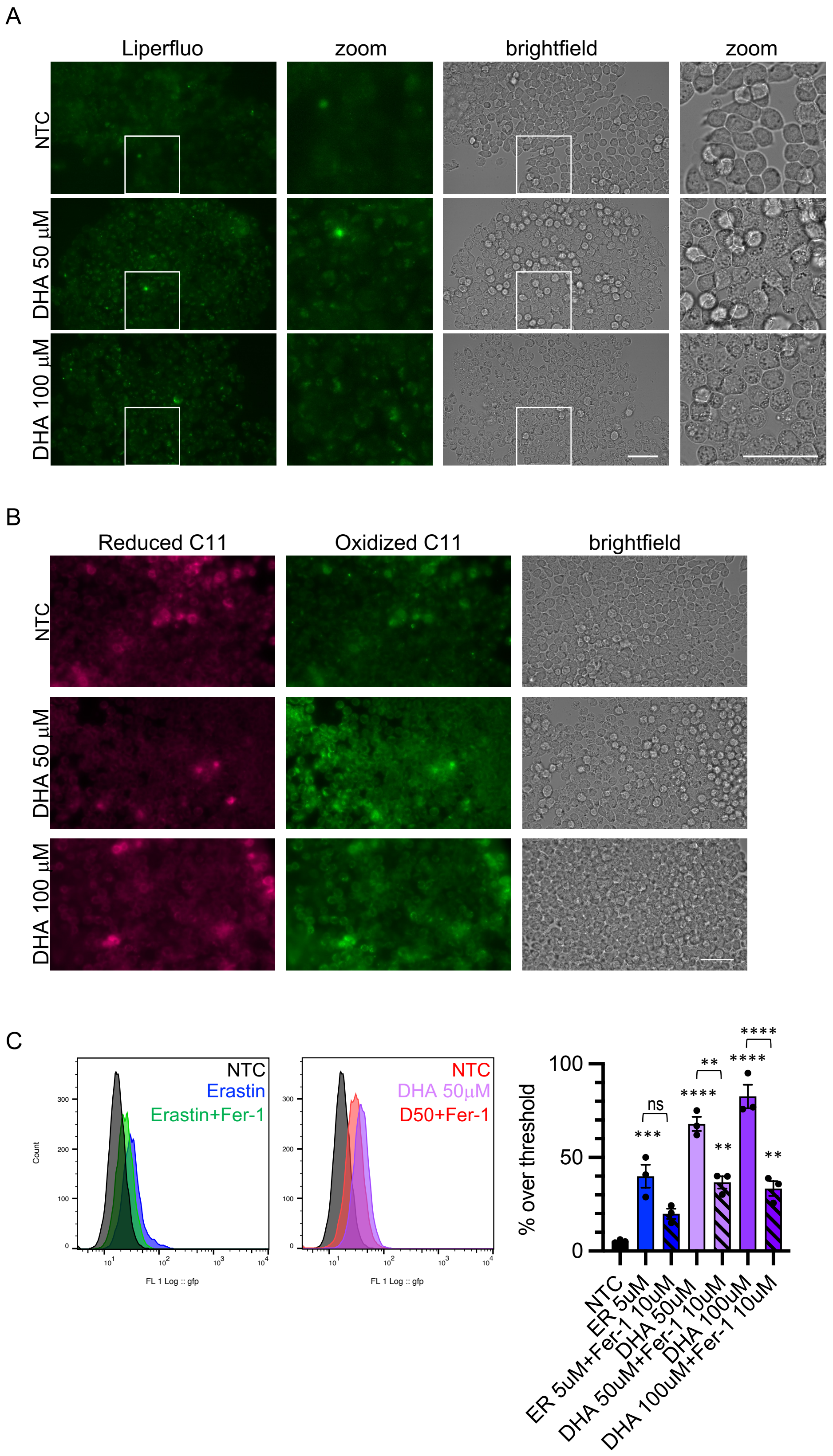

A
Liperfluo
zoom
brightfield
zoom
NTC
DHA 50 µM
DHA 100 µM
B
Reduced C11
Oxidized C11
brightfield
NTC
DHA 50 µM
DHA 100 µM
C
NTC
Erastin
Erastin+Fer-1
Count
FL 1 Log :: gfp
NTC
DHA 50µM
D50+Fer-1
FL 1 Log :: gfp
% over threshold
100
50
0
ns
**
****
***
****
**
****
**
**
NTC
ER 5uM
ER 5uM+Fer-1 10uM
DHA 50uM
DHA 50uM+Fer-1 10uM
DHA 100uM
DHA 100uM+Fer-1 10uM

Figure S2

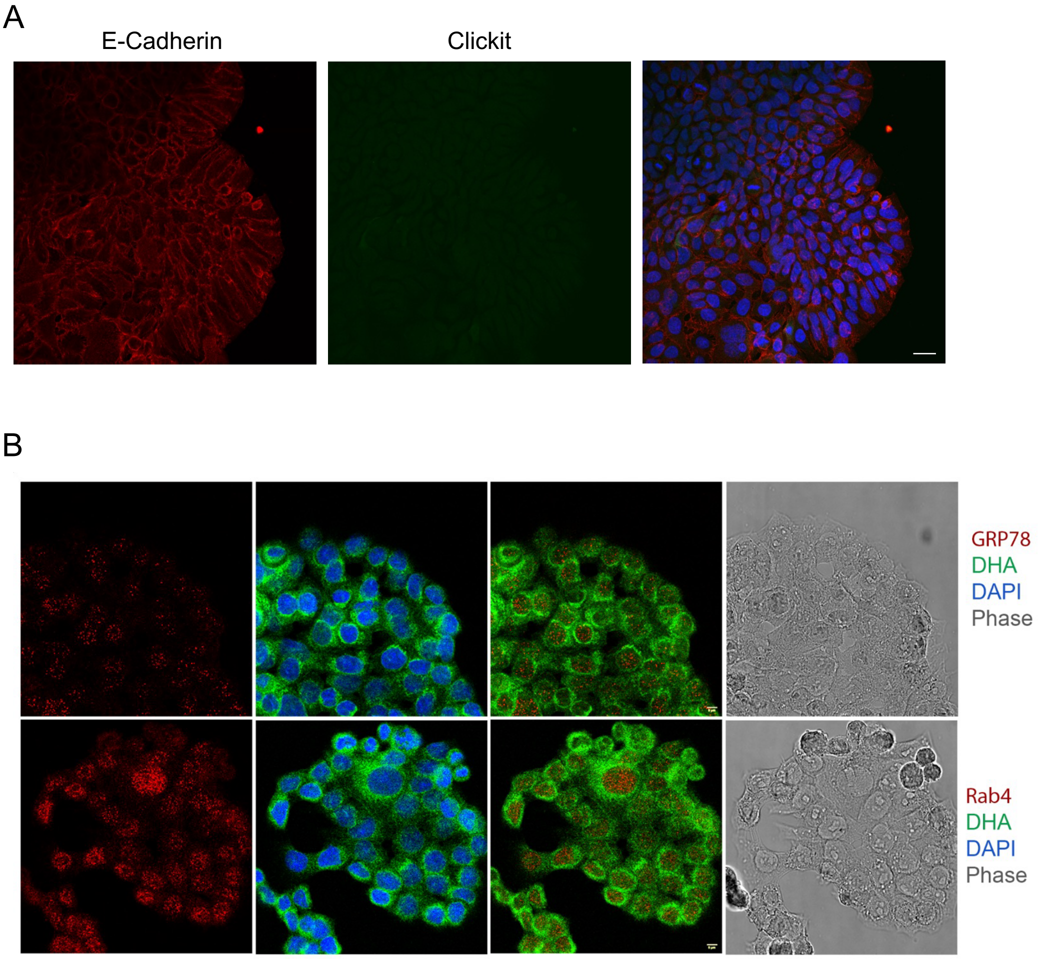
A
E-Cadherin
Clickit
B
GRP78
DHA
DAPI
Phase
Rab4
DHA
DAPI
Phase

Figure S3

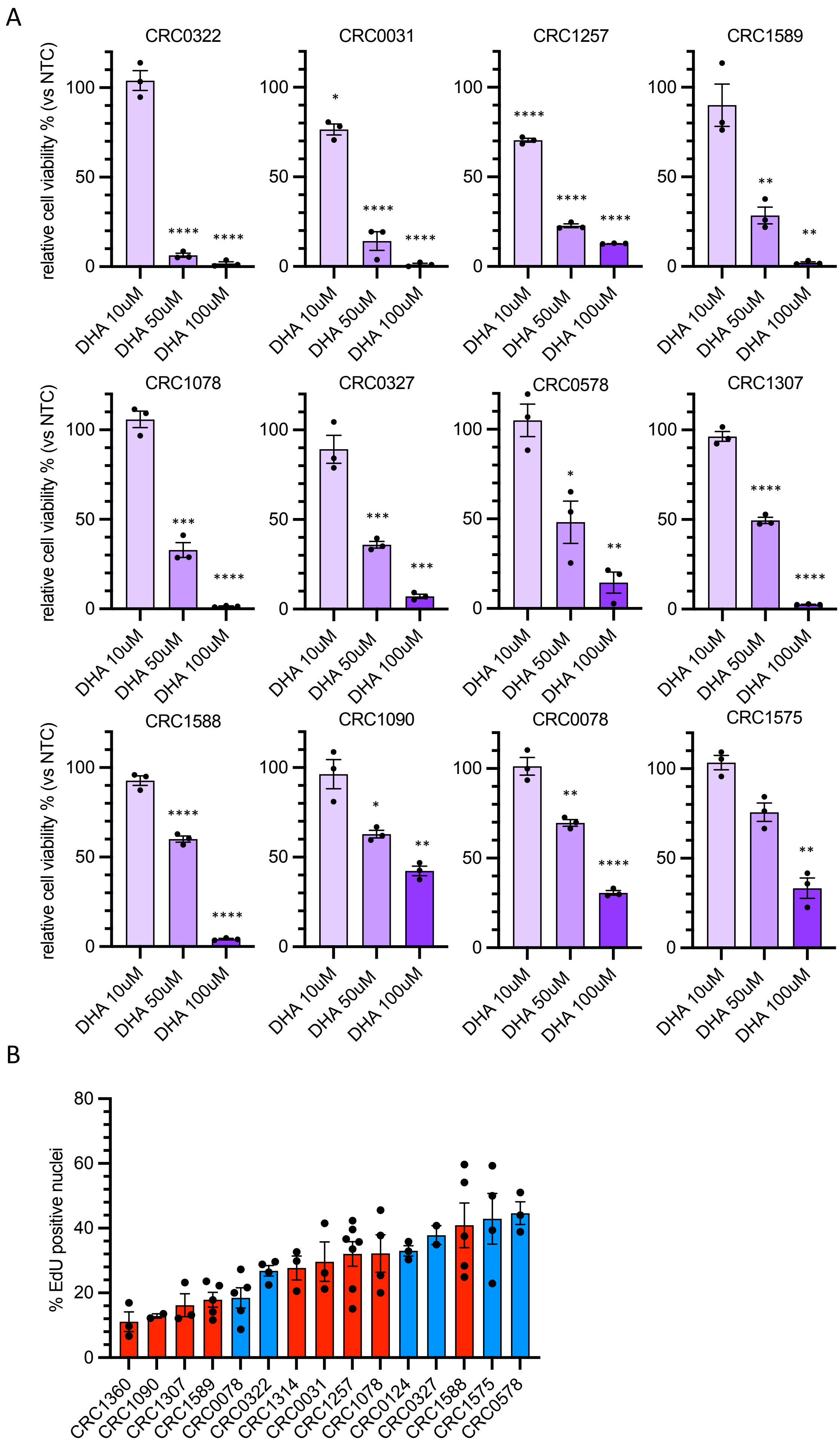
A
CRC0322
CRC0031
CRC1257
CRC1589
CRC1078
CRC0327
CRC0578
CRC1307
CRC1588
CRC1090
CRC0078
CRC1575
relative cell viability % (vs NTC)
DHA 10uM
DHA 50uM
DHA 100uM
B
% EdU positive nuclei
CRC1360
CRC1090
CRC1307
CRC1589
CRC0078
CRC0322
CRC1314
CRC0031
CRC1257
CRC1078
CRC0124
CRC0327
CRC1588
CRC1575
CRC0578

Figure S4

A

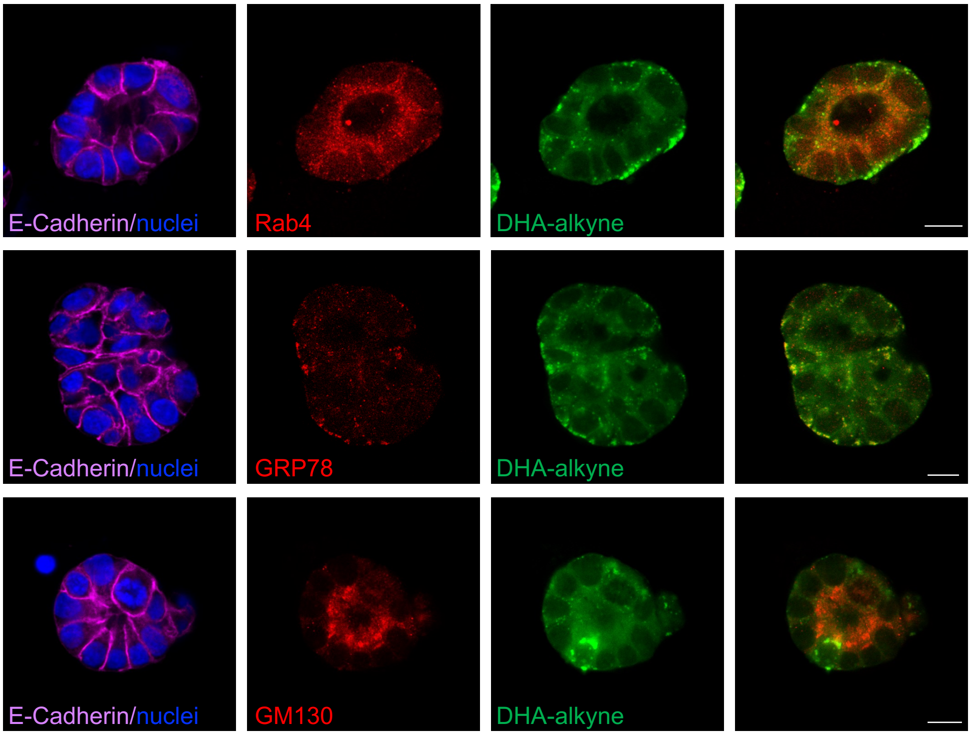

E-Cadherin/nuclei
Rab4
DHA-alkyne
E-Cadherin/nuclei
GRP78
DHA-alkyne
E-Cadherin/nuclei
GM130
DHA-alkyne

Figure S5

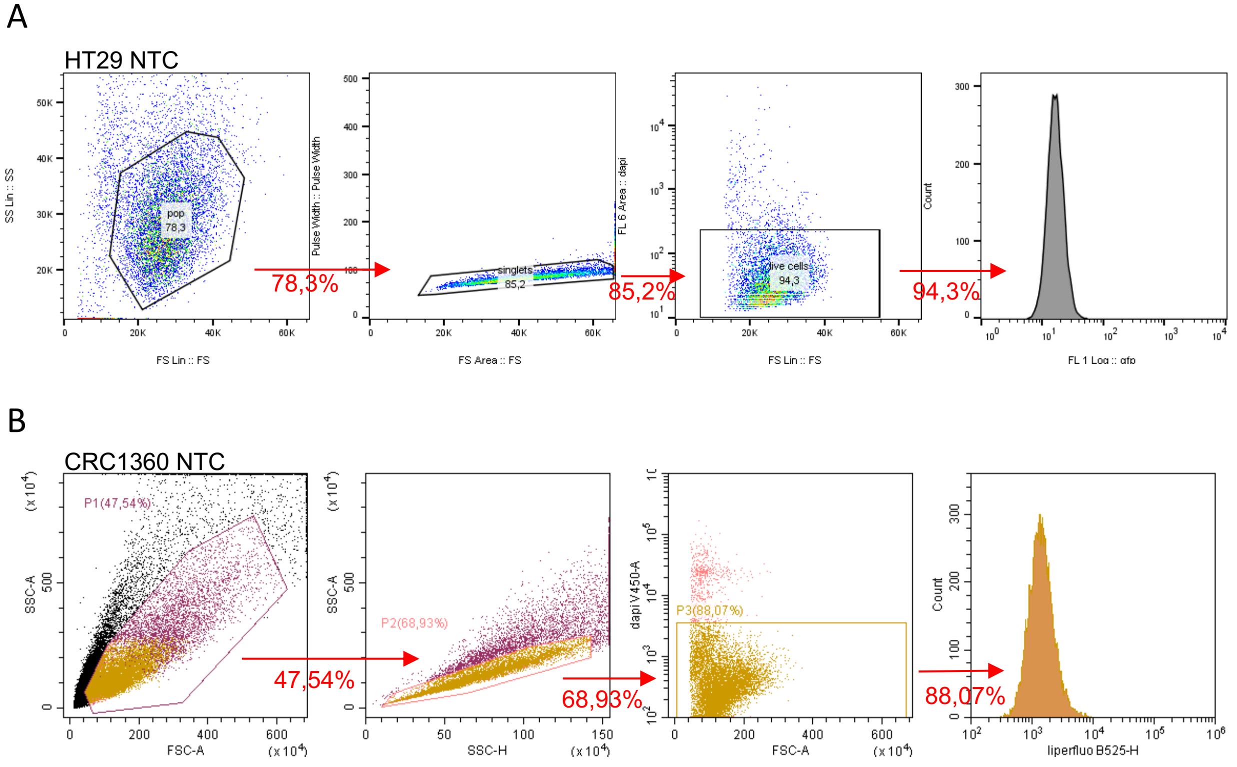
A
HT29 NTC
SS Lin :: SS
FS Lin :: FS
pop
78,3
78,3%
Pulse Width :: Pulse Width
FS Area :: FS
singlets
85,2
85,2%
FL 6 Area :: dapi
live cells
94,3
94,3%
Count
FL 1 Log :: gfp
B
CRC1360 NTC
P1(47,54%)
SSC-A
FSC-A
47,54%
P2(68,93%)
SSC-H
68,93%
dapi V450-A
P3(88,07%)
88,07%
Count
liperfluo B525-H

Figure S6
